# Development and external validation of a clinical prognostic score for death in visceral leishmaniasis patients in a high HIV co-infection burden area in Ethiopia

**DOI:** 10.1371/journal.pone.0178996

**Published:** 2017-06-05

**Authors:** Charles Abongomera, Koert Ritmeijer, Florian Vogt, Jozefien Buyze, Zelalem Mekonnen, Henok Admassu, Robert Colebunders, Rezika Mohammed, Lutgarde Lynen, Ermias Diro, Johan van Griensven

**Affiliations:** 1 Médecins Sans Frontières, Abdurafi, Ethiopia; 2 Department of Clinical Sciences, Institute of Tropical Medicine, Antwerp, Belgium; 3 Public Health Department, Médecins Sans Frontières, Amsterdam, The Netherlands; 4 Department of Internal Medicine, University of Gondar, Gondar, Ethiopia; Ohio State University, UNITED STATES

## Abstract

**Background:**

In Ethiopia, case fatality rates among subgroups of visceral leishmaniasis (VL) patients are high. A clinical prognostic score for death in VL patients could contribute to optimal management and reduction of these case fatality rates. We aimed to identify predictors of death from VL, and to develop and externally validate a clinical prognostic score for death in VL patients, in a high HIV co-infection burden area in Ethiopia.

**Methodology/Principal findings:**

We conducted a retrospective cohort study in north west Ethiopia. Predictors with an adjusted likelihood ratio ≥1.5 or ≤0.67 were retained to calculate the predictor score. The derivation cohort consisted of 1686 VL patients treated at an upgraded health center and the external validation cohort consisted of 404 VL patients treated in hospital. There were 99 deaths in the derivation cohort and 53 deaths in the external validation cohort. The predictors of death were: age >40 years (score +1); HIV seropositive (score +1); HIV seronegative (score -1); hemoglobin ≤6.5 g/dl (score +1); bleeding (score +1); jaundice (score +1); edema (score +1); ascites (score +2) and tuberculosis (score +1). The total predictor score per patient ranged from -1 to +5. A score of -1, indicated a low risk of death (1.0%), a score of 0 an intermediate risk of death (3.8%) and a score of +1 to +5, a high risk of death (10.4–85.7%). The area under the receiver operating characteristic curve was 0.83 (95% confidence interval: 0.79–0.87) in derivation, and 0.78 (95% confidence interval: 0.72–0.83) in external validation.

**Conclusions/Significance:**

The overall performance of the score was good. The score can enable the early detection of VL cases at high risk of death, which can inform operational, clinical management guidelines, and VL program management. Implementation of focused strategies could contribute to optimal management and reduction of the case fatality rates.

## Introduction

Visceral leishmaniasis (VL) or kala-azar is a protozoan infection caused by the *Leishmania donovani* species complex [1]. In East Africa and the Indian subcontinent, it is caused by *Leishmania donovani* and transmitted anthroponotically, through the bite of the female sandfly [2]. VL typically occurs in remote areas, characterized by limited access to health care and shortage of health professionals [2]. It is present in approximately 70 countries, with 200,000–400,000 new cases and 20,000–40,000 deaths occurring annually. Ethiopia is one of the top six high burden countries, with approximately 3.2 million people at risk and 3400–5000 VL cases occurring annually [3–5].

The risk factors for developing VL are for instance HIV, migration of non-immune people to endemic areas, young age, malnutrition and poor housing [6–8]. In Ethiopia, more than 60% of VL transmission and burden occurs in the north western semi-arid lowlands. In this VL focus, VL-HIV co-infection rate is 20–40%, the highest in the world and non-immune young male seasonal migrant workers from the highlands are the most at risk [4,6].

The main symptoms and signs of VL are fever, weight loss and organomegaly, and without treatment it is fatal [9]. Sodium stibogluconate (SSG) which is often given in combination with paromomycin (PM), is one of the main drugs for VL treatment in East Africa, however, SSG may cause severe adverse events (cardiotoxicity, nephrotoxicity, hepatotoxicity, pancreatitis) which can be fatal [10,11]. Liposomal amphotericin B (AmBisome), the alternative drug, is safer but expensive–therefore it should be administered to patients at most need, such as those with severe VL [12]. In 2014, the VL case fatality rate in Ethiopia was 2.6% [5]. However, differences in case fatality rates among subgroups have been documented. Studies show high case fatality rates among HIV co-infected (7.0–17.4%)[13,14], elderly (12.3%) [15] and malnourished (6.1%) [15] patients.

In East Africa, several predictors of death in VL patients have been identified, such as age, duration of illness, HIV serostatus, spleen size, nutritional status, hemoglobin level, bleeding, jaundice, weakness and tuberculosis (TB) [14–19]. However, no validated clinical prognostic score is currently available to predict death in clinical practice or VL programs. The optimal management and classification of VL severity remains poorly defined, and is highly variable across physicians and treatment sites [12,20,21].

A critical factor that could contribute to the optimal management and reduction of case fatality rates is the availability of evidence-based clinical prognostic tools [22]. Such tools are increasingly used in stratified or risk-based medicine, to identify the individuals requiring close observation and additional testing or treatment [23,24]. On the other hand, those with an excellent prognosis might be treated in an ambulatory way or at a decentralized level [23,24]. For instance, clinical prognostic tools or prediction scores relying on easy to measure clinical and laboratory information have been developed to predict death or morbidity in HIV infected patients [25].

A clinical prognostic score enabling the early detection of VL cases at high risk of death, can inform policy, clinical management guidelines and VL program management. In this study, we aimed to identify predictors of death from VL, and to develop and externally validate a clinical prognostic score for death in VL patients in a high HIV co-infection burden area in Ethiopia.

## Methods

### Study setting

The study was conducted in the Amhara region, north west Ethiopia. Development of the score was conducted at the Abdurafi health center supported by Médecins Sans Frontières (MSF). The health center is located in Abdurafi town, West-Armacheo district–a poor and remote district, with poor access to health care. The health center is upgraded, with a 100 bed capacity, emergency services (such as blood transfusion, oxygen therapy etc.) and capacity to treat VL and HIV co-infected patients.

External validation of the score was conducted at the Leishmania Research and Treatment Center (LRTC) at the University of Gondar Hospital supported by the Drugs for Neglected Diseases Initiative (DND*i*). The LRTC is located in Gondar city and is the main referral facility for critically ill or complicated VL cases. The main focus for both treatment centers, is the clinical management of VL and concomitant infections. They are the main VL treatment sites in the high HIV co-infection burden area and medical services are free of charge.

### Study design and population

We conducted a retrospective cohort study using routine program data collected using standardized data collection forms. To develop the score, we included all patients diagnosed with VL in Abdurafi health center between January 2008 and December 2013, whose outcome was cure or in-health center death. We excluded patients if their outcome was transferred-out, defaulted or not reported. To externally validate the score, we included all patients diagnosed with VL at the LRTC between January 2011 and December 2012, whose outcome was cure or in-hospital death. We excluded patients if their outcome was defaulted, treatment failure or not reported.

### Visceral leishmaniasis diagnosis

VL diagnosis was according to WHO guidelines [12]. Patients with the VL clinical description (prolonged fever, splenomegaly and wasting) from an endemic area or with a travel history to an endemic area, underwent further diagnostic evaluations. Patients with no prior VL treatment history (primary VL), were evaluated using a sequential testing algorithm including serology and parasitological testing. They were first screened using the rK39 rapid diagnostic test (IT-Leish^**®**^, Bio-Rad laboratories, USA) [26] and a positive test confirmed VL. In Abdurafi health center, patients testing negative were subsequently tested with the leishmania direct agglutination test (DAT) (Royal Tropical Institute, Amsterdam, The Netherlands) [27] and a high DAT titer (≥ 1:3200) confirmed VL. Patients with an intermediate DAT titer (1:800–1:1600) were subsequently diagnosed parasitologically. At the LRTC, rK39 negative patients underwent tissue aspiration (spleen, bone marrow or lymph node) and VL was confirmed parasitologically. At both treatment centers, patients with prior VL treatment history (relapse VL) were diagnosed parasitologically. A purely clinical diagnosis of relapse VL was made in patients with contra-indications for tissue aspiration (severe anemia, bleeding tendency, pregnancy or collapse). Patients fulfilling the VL clinical description but with negative serological and/or parasitological test results were evaluated for other conditions compatible with their clinical presentation. If no alternative diagnosis was made or the condition persisted after treatment of an alternative diagnosis, patients were rescreened for VL, one to two weeks later.

### Visceral leishmaniasis treatment

VL treatment was only administered during admission at the health facility.

#### Derivation cohort

VL disease severity was classified into severe and non-severe, according to MSF guidelines. This classification was based on an algorithm that combined different risk factors of death (weakness, age, body mass index and hemoglobin level)[16,20]. In 2008 to 2012, patients with non-severe primary VL were treated with SSG (Albert David Ltd., Kolkata) at dosages of 20 mg/kg/day (minimum daily dose 200 mg, no maximum dose) by intramuscular injection for a total duration of 30 days. In 2013, the treatment protocol was changed in line with the national guidelines, to the combination of SSG and PM (Gland Pharma Ltd., Hyderabad, India) at dosages of 20 mg/kg/day and 15 mg sulphate/kg/day (11 mg/base/kg/day) respectively by intramuscular injection for a total duration of 17 days [28]. Those with severe primary VL, relapse VL and VL-HIV coinfection were treated with liposomal amphotericin B (AmBisome, Gilead Sciences) at a total dose of 30 mg/kg divided into 6 infusions of 5 mg/kg on alternate days. In 2011, the first line treatment for VL-HIV was changed to a combination therapy of AmBisome at the above dosage and miltefosine (Impavido, Paladin Labs, Montreal, Canada) administered orally for 28 days (100 mg/day in patients weighing more than 25 kg and 50 mg per day in those that were 25 kg or less).

#### Validation cohort

VL disease severity was classified as non-severe and severe, based on clinical judgement [12]. Non-severe primary VL and the majority of severe primary VL, relapse VL and VL-HIV patients received SSG monotherapy at the same dosage as in the derivation cohort. When AmBisome was available, some severe primary VL, relapse VL and VL-HIV patients, received AmBisome monotherapy at the same dosage as in the derivation cohort.

### Visceral leishmaniasis treatment outcomes

Only VL treatment outcomes that occurred during admission at the health facility were documented. There was no patient follow-up after exit from the health facility. In-health center/in-hospital death were defined as death during VL treatment at the health facility. Cure was defined as improvement in symptoms and signs of VL, 17–30 days after treatment initiation (i.e. absence of fever, decrease in spleen size, increase in hemoglobin, weight gain) and a negative parasitological test in VL relapse patients or those with poor treatment response. Transfer-out was defined as referral to another health facility for any reason. Defaulting was defined as absconding from treatment. Treatment failure was defined as a positive parasitological test at the end of treatment.

### Data collection and measurement of variables

From the VL program onset, clinical data were collected using standardized data collection tools and stored in electronic databases. The databases were updated on a daily basis by data managers. The data were collected at admission through history taking, clinical examination, laboratory and/or radiological investigations, and treatment prescriptions (VL regimen).

The following variables were assessed from patient history: age (years), sex, residential status (migrant worker, settler and resident), and the duration of illness (months). While the following were assessed by clinical examination: weight (kilograms), height (meters)/length (centimeters), jaundice, ascites, spleen size (centimeters), bleeding, edema and the level of weakness. Anthropometric parameters were calculated according to WHO guidelines [29–31] [weight-for-length/height z-score in patients 6 months–5 years; body mass index (BMI)-for-age z-score in patients 5–19 years; BMI [weight in kilogram–(height in meter)^2^] in patients >19 years]. In different age groups, severe malnutrition was defined as follows: >19 years (BMI <16.0 kg/m^2^); 5–19 years (BMI-for-age z-score <–3); <5 years (weight-for-length/height z-score <–3). The spleen size (centimeters) was measured from the junction of the anterior axillary line and the left coastal margin to the tip of the spleen. In the derivation cohort, weakness severity was defined according to MSF guidelines [20] as follows: [State of collapse (in adults/older children: unable to sit up unaided and cannot drink unaided. In babies: floppy when held in arms and unable to feed unaided); severely weak (in adults/older children: cannot walk 5 meters without assistance and in babies: unable to sit upright unaided); other types of weakness were classified as “other”]. In the external validation cohort, weakness severity was classified as present or absent, based on clinical judgement.

The following variables were assessed by laboratory and/or radiological investigations. HIV testing was based on MSF and national rapid diagnostic testing algorithms. In Abdurafi Health center, a positive test was defined by two positive serological tests performed in parallel {KHB (Shanghai Kehua Bio-engineering Co-Ltd, Shanghai, China) and STAT-PAK ^TM^ (Chembio HIV1/2, Medford, New York, USA)} and confirmed by the ELISA test {ImmunoComb (Orgenics ImmunoComb® II, HIV 1&2 Combfirm)}. At the LRTC, a positive test was defined by two sequential positive serological tests; KHB followed by STAT-PAK ^TM^ and in case of discrepancy, a tie-breaker test Uni-Gold (Trinity Biotech PLC, Bray, Ireland) was used. TB was diagnosed according to WHO guidelines [32]. Hemoglobin level in grams per deciliter (g/dl) was determined using a hematology analyzer–Beckman Coulter A^c^T diff, Beckman Coulter Inc., 2003, USA.

### Sample size

To develop the score, we aimed for 10 deaths per variable in the final model. In external validation, the sample size was pre-determined by the available data (53 deaths) [22].

### Data analysis

The main outcome measured was death. The choice of variables analyzed were based on literature review for predictors of death [14–19] and the number of variables in our dataset, that when included in the model would ensure a favorable ratio of deaths per variable [22]. Variables assessed as possible predictors of death were: age, nutritional status, jaundice, relapse status (primary VL or relapse VL), duration of illness, TB, HIV serostatus, hemoglobin level, ascites, spleen size, bleeding, edema, weakness and VL treatment regimen. The score was developed using the Spiegelhalter and Knill-Jones method [33,34].

The whole data set was used to develop the score. Five-fold cross validation and external validation were performed to evaluate the performance of the score [35]. Other than “age” which was categorized based on information from the literature, continuous variables were dichotomized as guided by receiver operating characteristic curves (ROC), with the optimal cut-off at the point with the highest sum of sensitivity and specificity. The cut-offs were rounded to values that are easy to use in clinical practice. Differences in proportions of categorical variables between the derivation and external validation cohorts were compared using Fisher exact or Chi-squared tests.

The score was built as follows: crude likelihood ratios (LHR) were calculated for all predictors and those with a LHR ≥2 or ≤0.5 were selected for use in the next step. To adjust for correlations between predictors, LHR were adjusted using multiple logistic regression. Variables with adjusted LHR ≥1.5 or ≤0.67 were then selected and the model was refitted. This procedure was repeated until all selected variables had an adjusted LHR ≥1.5 or ≤0.67. To develop the scoring system, the score for each predictor was obtained by calculating the natural logarithm of the adjusted LHR (a value of 0 was allocated to missing data) and rounding this result to the nearest integer. Summing the scores of the individual’s predictors yielded the total score for each patient. The observed probability of death by prognostic score was then calculated. The performance of the score was evaluated by calculating the sensitivity, specificity, positive predictive value (PPV) and negative predictive value (NPV) at different cut-offs. Its overall performance was assessed using the area under the receiver operating characteristic curve (AUROC) and 95% confidence intervals (CI). An AUROC of 0.5 would imply no discrimination, 0.7–<0.8 would imply acceptable discrimination, 0.8–<0.9 would imply excellent discrimination and ≥0.9 would imply outstanding discrimination [36]. Statistical analysis was done using Stata 14 software.

### Ethics approval

Ethics approval was received from the Institutional Review Board of the Institute of Tropical Medicine, Antwerp, Belgium, the Ethical Review Board of the Institute of Public Health, Gondar University, Ethiopia and as this research was an anonymous retrospective data analysis of routine program data, patient written informed consent was not necessary and it was exempted from full formal ethical approval as per MSF International Ethics Review Committee criteria. It was conducted with permission from the Medical Director of the MSF Operational Centre Amsterdam.

## Results

### Derivation cohort

**Fig 1:**
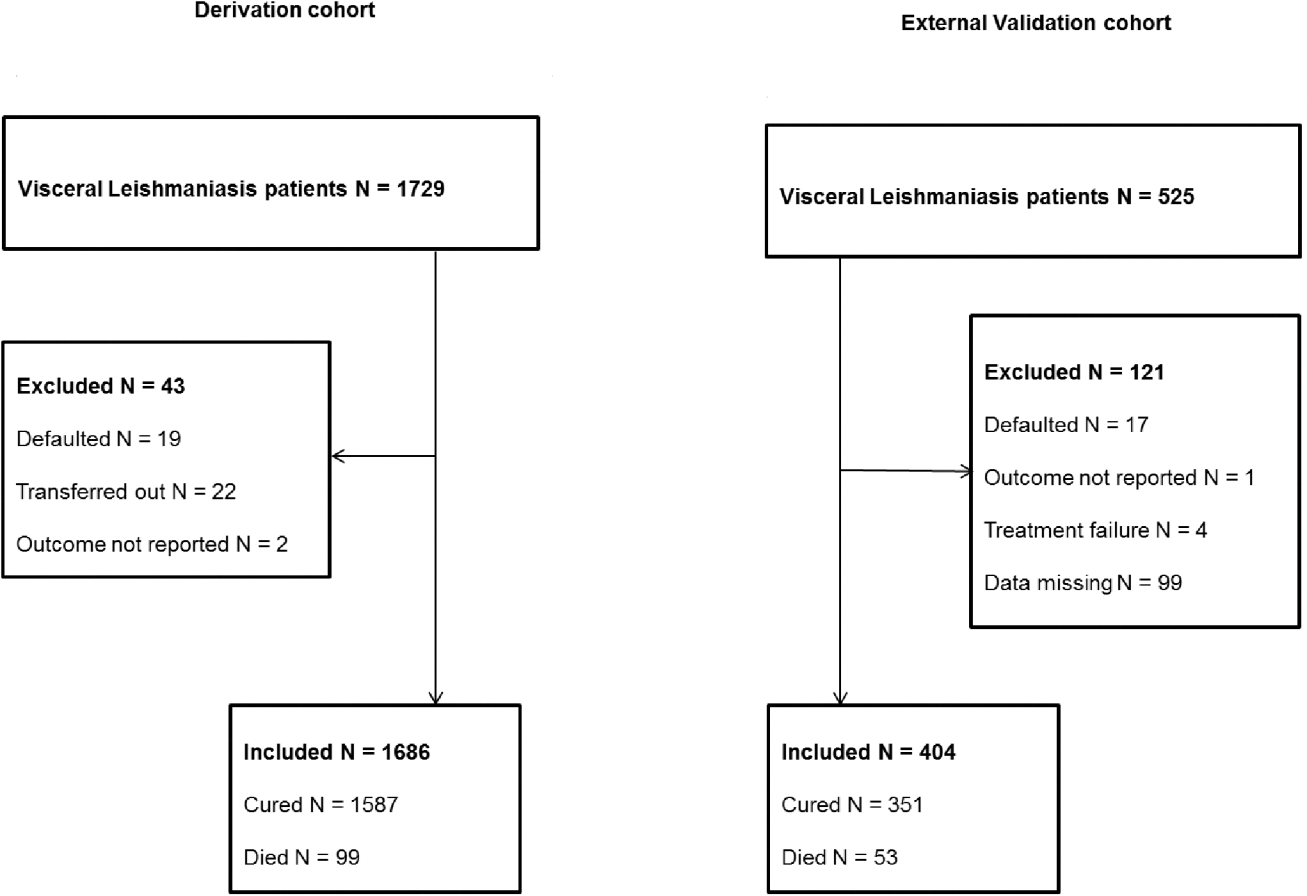
Flow diagram showing the number of patients in the study and their outcomes.

Between January 2008 and December 2013, 1729 patients were diagnosed with VL and treated at the Abdurafi health center. Nineteen patients defaulted during treatment, 22 were transferred out and 2 had unknown treatment outcome. These 43 patients (2.5%) were excluded from the study. A total of 1686 patients were included in the study and 99 of them died (Fig 1).

### External validation cohort

Between January 2011 and December 2012, 525 patients were diagnosed with VL and treated at the LRTC. Seventeen patients defaulted during treatment, 4 had treatment failure, 2 had unknown treatment outcome and 99 had missing data. These 121 patients (23.0%) were excluded from the study. A total of 404 patients were included in the study and 53 of them died (Fig 1).

### Patient characteristics in the development and external validation cohorts

In the derivation and external validation cohorts respectively, the majority of patients were young (median age 23 (IQR 20–28) years *vs*. 25 (IQR 20–28) years), male (95.9% *vs*. 97.5%) and migrant workers (63.8% *vs*. 80.9%). In the external validation cohort compared to the derivation cohort, there was a higher proportion of patients with the following characteristics: hemoglobin ≤6.5g/dl (30.2% *vs*. 22.4%); bleeding (28.7% *vs*. 3.4%); jaundice (16.1% *vs*. 2.6%); edema (28.2% *vs*. 7.8%) and ascites (30.2% *vs*. 1.3%). VL-HIV co-infection rates were high in both cohorts: 19.3% in the derivation cohort and 13.6% in the external validation cohort. A higher proportion of patients in the derivation cohort, were treated with an initial VL regimen containing AmBisome (37.0% *vs*. 8.2%), and the case fatality rate was lower in the derivation cohort (5.9% *vs*. 13.1%) (Table 1).

**Table 1:** Comparison of patient characteristics in the development and external validation cohorts.

Characteristic	Derivation cohort (N = 1686)	External validation cohort (N = 404)	*P*
**Age (years), median (IQR)**	23 (20–28)	25 (20–28)	
**Age groups (years), n (%)**^**1**^			
- <5	40 (2.3)	0 (0.0)	<0.001^3^
- 5–18	264 (15.7)	39 (9.7)	
- >18–40	1305 (77.4)	348 (86.1)	
- >40	77 (4.6)	15 (3.7)	
- Missing	0 (0.0)	2 (0.5)	
**Sex, n (%)**			
- Male	1617 (95.9)	394 (97.5)	0.15^3^
- Female	68 (4.0)	10 (2.5)	
- Missing	1 (0.1)	0 (0.0)	
**Residency status, n (%)**			
- Resident	451 (26.8)	77 (19.1)	<0.001^3^
- Migrant worker	1077 (63.8)	327 (80.9)	
- Settler	156 (9.3)	0 (0.0)	
- Missing	2 (0.1)	0 (0.0)	
**Duration of illness (months), median (IQR)**	1 (1–2)	2 (1–3)	
**Duration of illness ≥2 months, n (%)**			
- Yes	528 (31.3)	209 (51.7)	<0.001^4^
- No	1132 (67.1)	195 (48.3)	
- Missing	26 (1.6)	0 (0.0)	
**Relapse VL, n (%)**			
- Yes	156 (9.2)	23 (5.7)	0.02^4^
- No	1530 (90.8)	381 (94.3)	
**HIV serostatus, n (%)**			
- Positive	326 (19.3)	55 (13.6)	0.003^3^
- Negative	1319 (78.2)	349 (86.4)	
- Discordant	11 (0.7)	0 (0.0)	
- Missing	30 (1.8)	0 (0.0)	
**Spleen size (cm), median (IQR)**	6 (4–10)	8 (5–11)	
**Spleen size ≥11 cm**			
- Yes	285 (16.9)	115 (28.5)	<0.001^4^
- No	1379 (81.8)	285 (70.5)	
- Missing	22 (1.3)	4 (1.0)	
**Severe malnutrition, n (%)**			
- Yes	554 (32.9)	136 (33.7)	<0.001^4^
- No	1021 (60.5)	165 (40.8)	
- Missing	111 (6.6)	103 (25.5)	
**Hemoglobin level (g/dl): median (IQR)**	8.1 (6.7–9.8)	7.8 (6.3–9.4)	
**Hemoglobin level ≤6.5 g/dl, n (%)**			
- Yes	378 (22.4)	122 (30.2)	<0.001^4^
- No	1284 (76.2)	280 (69.3)	
- Missing	24 (1.4)	2 (0.5)	
**Bleeding, n (%)**			
- Yes	57 (3.4)	116 (28.7)	<0.001^3^
- No	1622 (96.2)	288 (71.3)	
- Missing	7 (0.4)	0 (0.0)	
**Jaundice, n (%)**			
- Yes	44 (2.6)	65 (16.1)	<0.001^3^
- No	1508 (89.4)	339 (83.9)	
- Missing	134 (8.0)	0 (0.0)	
**Weakness, n (%)**			
**Classification at Abdurafi health center**			
- Collapse	4 (0.2)	-	
- Severe	295 (17.5)	-	
- Other	1381 (81.9)	-	
- Missing	6 (0.4)	-	
**Classification at LRTC**			
- Yes	-	374 (92.6)	
- No	-	30 (7.4)	
- Missing	-	0 (0.0)	
**Edema, n (%)**			
- Yes	132 (7.8)	114 (28.2)	<0.001^4^
- No	1245 (73.9)	290 (71.8)	
- Missing	309 (18.3)	0 (0.0)	
**Ascites, n (%)**			
- Yes	21 (1.3)	122 (30.2)	<0.001^3^
- No	1356 (80.4)	282 (69.8)	
- Missing	309 (18.3)	0 (0.0)	
**Tuberculosis, n (%)**			
- Yes	120 (7.1)	10 (2.5)	<0.001^3^
- No	1565 (92.8)	394 (97.5)	
- Missing	1 (0.1)	0 (0.0)	
**VL treatment regimen**^**2**^**, n (%)**			
- AmBisome based initial regimen	624 (37.0)	33 (8.2)	<0.001^4^
- SSG based initial regimen	1060 (62.9)	369 (91.3)	
- Missing	2 (0.1)	2 (0.5)	
**VL outcome, n (%)**			
- Died	99 (5.9)	53 (13.1)	<0.001^4^
- Cured	1587 (94.1)	351 (86.9)	

IQR–Inter Quartile Range; VL–Visceral leishmaniasis; LRTC–Leishmania Research and Treatment Center; SSG–Sodium stibogluconate

^**1**^ All percentages (%) are column percentages

^**2**^ The first VL treatment regimen.

^**3**^ Fisher’s exact test.

^**4**^ Chi-squared test.

### Predictors of death

The predictors of death were: age >40 years, HIV seropositive, HIV seronegative, hemoglobin ≤6.5 g/dl, bleeding, jaundice, edema, ascites and TB. The total predictor score per patient ranged from –1 to +5 (Table 2).

**Table 2:** Number of deaths in the derivation cohort, likelihood ratios for predicting death and score by predictor.

Predictor	Number of deaths*, (%)^1^	Crude LHR	Adjusted LHR^2^	Score
**Total**	99 (5.9)			
**Age groups (years)**^**3**^				
- <5	4 (10.0)	1.96	–	–
- 5–18	3 (1.1)	0.98	–	–
- >18–40	77 (5.9)	1.00	–	–
- 5–18	3 (1.1)	0.99	–	–
- >40	15 (19.5)	3.94	2.22	+1
- 5–18	3 (1.1)	0.88	0.93	0
**Relapse VL**				
- Yes	20 (12.8)	2.38	–	–
- No	79 (5.2)	0.87	–	–
**Duration of illness ≥2 months**				
- Yes	35 (6.6)	1.20	–	–
- No	58 (5.1)	0.91	–	–
**HIV serostatus**				
- Positive	51 (15.6)	3.20	3.04	+1
- Negative	39 (3.0)	0.53	0.54	^_^1
**Severe malnutrition**				
- Yes	46 (8.3)	1.78	–	–
- No	30 (2.9)	0.60	–	–
**Hemoglobin ≤6.5 g/dl**				
- Yes	47 (12.4)	2.29	2.16	+1
- No	50 (3.9)	0.65	0.67	0
**Spleen size ≥11 cm**				
- Yes	20 (7.0)	1.28	–	–
- No	74 (5.4)	0.94	–	–
**Bleeding**				
- Yes	9 (15.8)	3.13	3.11	+1
- No	89 (5.5)	0.93	0.93	0
**Jaundice**				
- Yes	11 (25)	5.58	3.21	+1
- No	78 (5.2)	0.89	0.93	0
**Weakness (severe/collapse)**				
- Yes	38 (12.7)	2.33	1.64	0
- No	61 (4.4)	0.74	0.84	0
**Edema**				
- Yes	22 (16.7)	3.35	2.36	+1
- No	56 (4.5)	0.78	0.84	0
**Ascites**				
- Yes	10 (47.6)	15.23	5.84	+2
- No	67 (4.9)	0.87	0.92	0
**Tuberculosis**				
- Yes	23 (19.2)	3.83	1.71	+1
- No	76 (4.9)	0.82	0.92	0
**VL treatment regimen**^**4**^				
- AmBisome based initial regimen	76 (12.2)	2.21	–	–
- SSG based initial regimen	23 (2.2)	0.36	–	–

VL–Visceral leishmaniasis; LHR–Likelihood ratio; SSG–Sodium stibogluconate

^*^ Data are missing when the total number of deaths for a predictor are less than 99.

^**1**^ All percentages (%) are row percentages.

^**2**^ Adjusted for all variables with an adjusted LHR ≥1.5 or ≤0.67 (age >40 years, HIV serostatus, hemoglobin ≤6.5 g/dl, bleeding, jaundice, edema, ascites and tuberculosis).

^**3**^ The reference age-group is 5–18 years; it is compared to age-groups, <5, 18–40 and >40 years.

^**4**^ The first VL treatment regimen.

**Fig 2:**
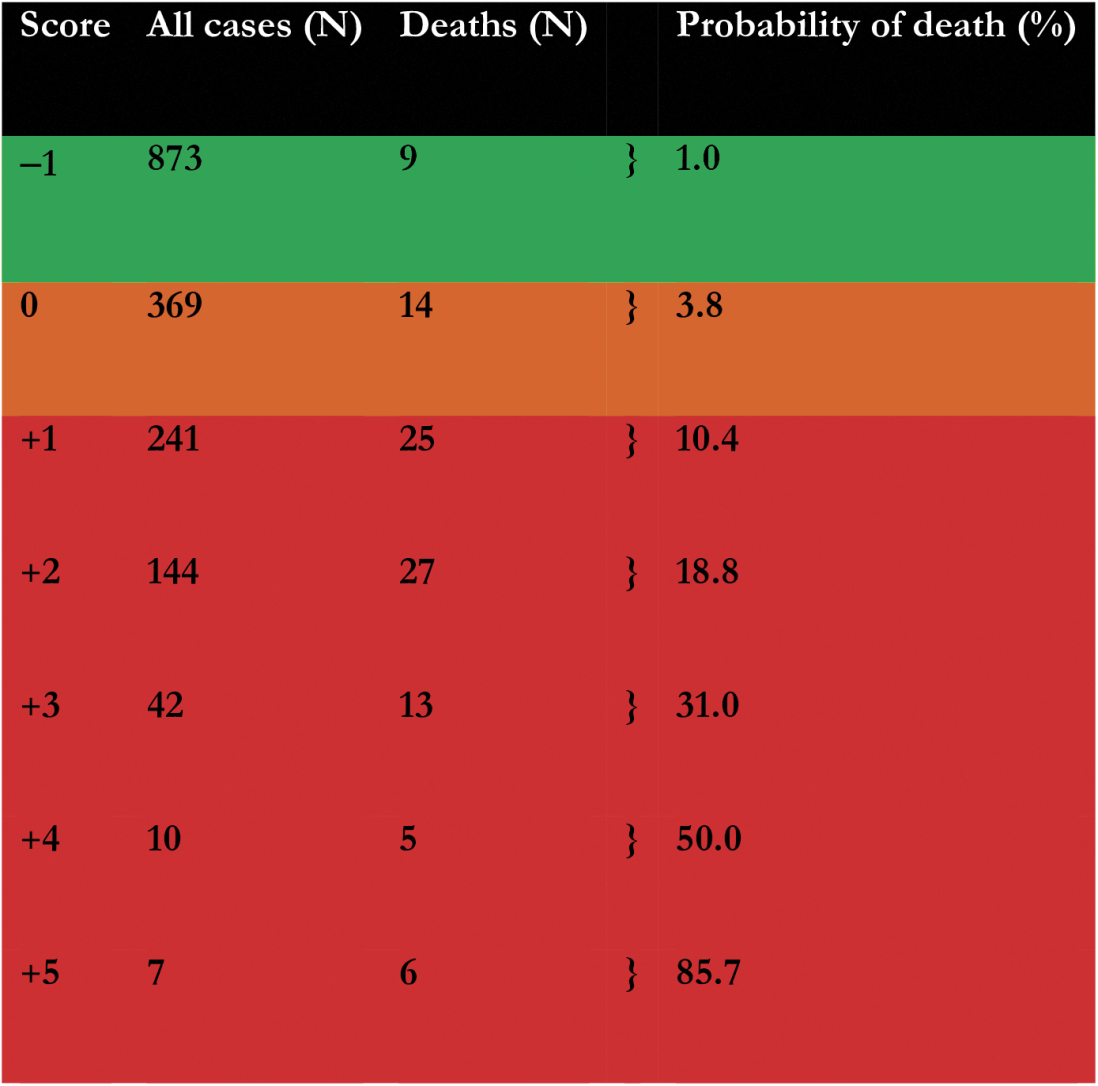
Probability of death by clinical prognostic score.

The probability of death ranged from 1.0% for patients with a score of –1 to 85.7% for those with a score of +5. Eight hundred and seventy three patients (51.8%) had a score of –1 and a low risk of death (1.0%), 369 patients (21.9%) had a score of 0 and an intermediate risk of death (3.8%), and 444 patients (26.3%) had a score of +1 to +5 and a high risk of death (10.4–85.7%) (Fig 2).

As the score cut-off increased, the sensitivity decreased from 90.9% to 6.1% while the specificity and PPV increased from 54.4% to 99.9%, and from 11.1% to 85.7%, respectively (Table 3).

**Table 3:** Diagnostic accuracy of the clinical prognostic score at different cut-offs.

Score	n^1^ (%)	Sensitivity	Specificity	PPV	NPV
**≥ 0**	90 (5.3)	90.9%	54.4%	11.1%	99.0%
**≥ 1**	76 (4.5)	76.8%	76.8%	17.1%	98.1%
**≥ 2**	51(3.0)	51.5%	90.4%	25.1%	96.8%
**≥ 3**	24 (1.4)	24.2%	97.8%	40.7%	95.4%
**≥ 4**	11 (0.7)	11.1%	99.6%	64.7%	94.7%
**≥ 5**	6 (0.4)	6.1%	99.9%	85.7%	94.5%

PPV–Positive predictive value; NPV–Negative predictive value

^1^Number of patients dead/overall number of visceral leishmaniasis patients, presented as a percentage.

### Validation of the prognostic score

**Fig 3:**
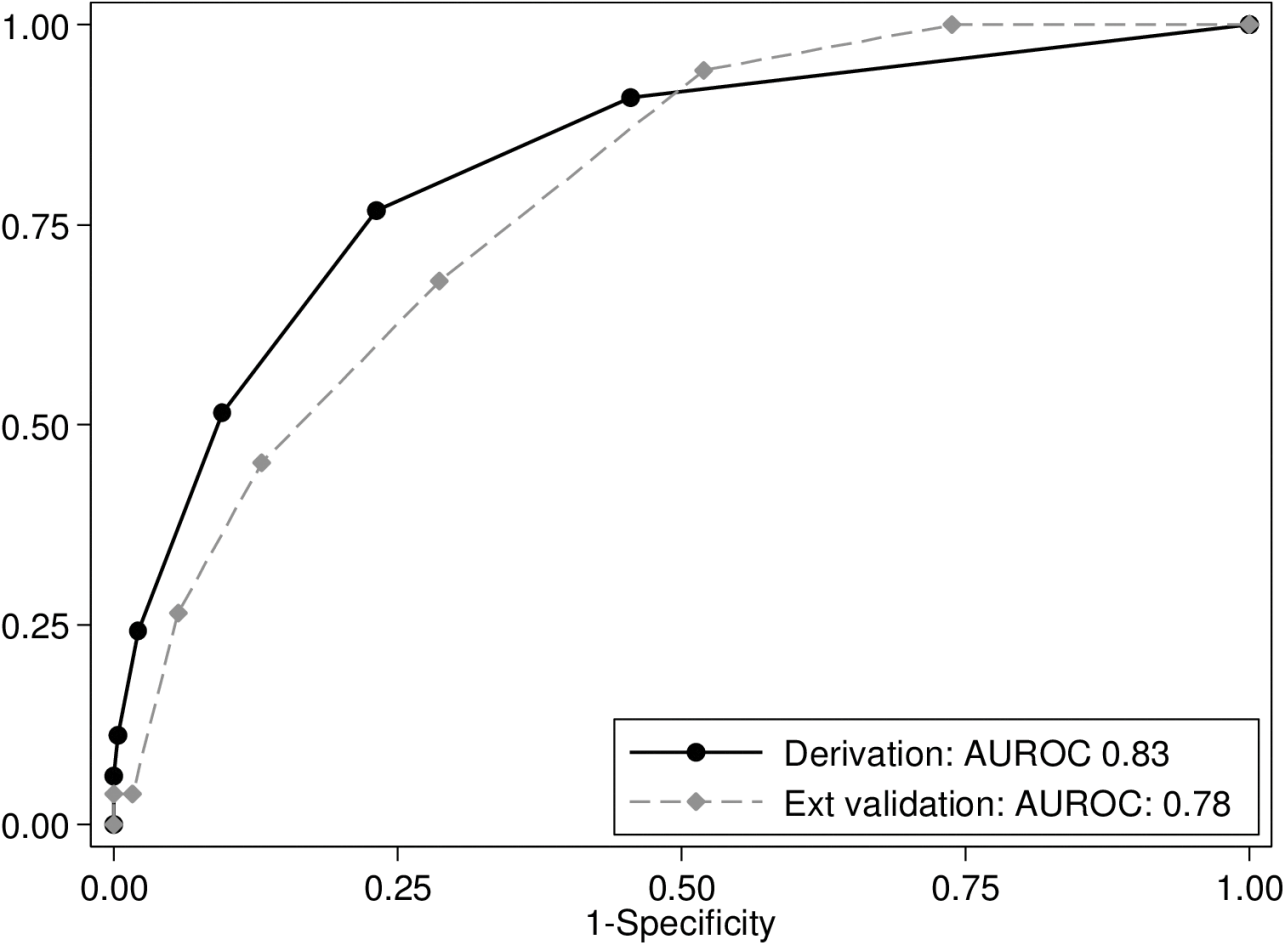
ROC curve summarizing the performance of the scoring system during development and external validation.

The AUROC was 0.83 (95% CI 0.79–0.87) in derivation, 0.82 (95% CI 0.77–0.88) in five-fold cross validation and 0.78 (95% CI 0.72–0.83) in external validation (Fig 3).

## Discussion

Using standardized VL program data from a high VL-HIV burden setting, we identified predictors of death in VL patients, and developed and externally validated a clinical prognostic score for death in VL patients, in a high HIV co-infection burden area in Ethiopia. The overall performance of the score was good with an AUROC of 0.83 (95% CI 0.79–0.87) in derivation and 0.78 (95% CI 0.72–0.83) in external validation. The AUROC is the best measure to assess the overall performance of a clinical score. Positive and negative predictive values also depend on the prevalence of the outcome and hence are context-dependent. In general, an AUROC >0.7 is considered clinically useful and we have used this value to evaluate the performance of our score [36]. For validation, we have used the same value, in addition to the drop in AUROC, relative to the derivation AUROC. The confidence intervals for derivation and validation AUROC overlap, suggesting a minimal drop in diagnostic performance and hence providing rather favorable findings on validation. As most of the predictors are easily identified by health professionals, the tool would be expected to be clinically relevant and easy to use in clinical practice.

In general, the patient characteristics in this study are similar to those reported in other studies from Ethiopia [6,14,17]. The difference in patient characteristics and case fatality rate at Abdurafi health center compared to the LRTC could be due to several reasons. The LRTC is the main referral facility for critically ill or complicated VL cases, and this is also supported by the data (Table 1), which shows higher proportions of patients with predictors of death. Additionally, due to limited availability of AmBisome, the majority of VL-HIV co-infected patients were treated with SSG which is known to increase mortality and this may have contributed to the high case fatality [37,38]. Other factors that could contribute to the lower case fatality rate at Abdurafi health center, is the support provided by MSF in running the VL related activities, including free access to comprehensive health care in a relatively resourced setting. Importantly, despite the significant differences in patient populations within the derivation and external validation cohorts (Table 1), the score performed well during external validation.

The predictors of death, identified in this study (age >40 years, HIV seropositive, HIV seronegative, hemoglobin ≤6.5 g/dl, bleeding, jaundice, edema, ascites and TB) are similar to those reported in other studies [6,14–19,39,40]. They indicate the role of VL-HIV co-infection, bone marrow suppression, splenic sequestration and late stage VL disease. HIV and *Leishmania* parasites infect and multiply within cells of myeloid or lymphoid origin. Both infections change the predominant cellular immune response of Th1 to Th2 through complex mechanisms mediated by cytokines increasing susceptibility to both infections [41,42]. Severe anemia arises and may cause congestive heart failure characterized by edema [43]. Bleeding may occur as a result of the systemic inflammatory response [44,45]. The state of severe immunosuppression that ensues, favors the development of opportunistic infections such as TB [14,18,46–48]. Patients aged >40 years could be at increased risk of death because of underlying co-morbidities (e.g. cardiovascular diseases), lower immunity, severe VL disease and/or severe SSG related adverse events [18,19,39,49,50]. Liver dysfunction with jaundice and ascites typically occurs in advanced VL disease [51,52]. In this study, ascites alone, had a score of +2 and a probability of death of 18.8% (Fig 2).

VL treatment varied across patients, with more severe cases more likely to get AmBisome, a safer drug. This also occurred in all reported VL clinical prediction scores, and many of the prediction scores for other diseases [22]. Nevertheless, VL treatment was not retained in the final score, probably because in the presence of major predictors of death, the predictive effect of treatment on outcome may be minimal [22]. In our study, the following variables were not predictors of death, contrary to what is reported in other studies: young age group (<5 years) [16,18], duration of illness ≥5 months [16,19], severe malnutrition [16,19] and splenomegaly ≥11 cm [16]. This is probably because these variables were present in a lower proportion of our patients or the cut-offs used in the other studies were more extreme (included a more ill cohort). Weakness was also not a predictor of death, possibly because of differences in case definitions, patient populations and the impact of treatment [17,22,50]. Only four patients had the highest grade of weakness (collapse), and all of them were cured. In an exploratory analysis, we found that VL relapse did not predict death.

The score can enable the early detection of VL cases at high risk of death, which can inform operational, clinical management guidelines and VL program management. Busy treatment programs can use this information to organize patient care according to different patient paths, with different levels of care. However, the decisions on how to use the score, and which cut-offs to apply for decision making require careful consideration as this is context-dependent and largely determined by operational factors. We present the diagnostic performance at different cut-offs, allowing the reader to decide on which cut-off to use in their setting. In relatively better resourced settings (eg. non-governmental organization settings), with sufficient human resources, a higher number of patients could receive closer monitoring/more intensive care. In less resourced settings (eg. overwhelmed public hospitals), applying the same cut-offs might not be feasible, and hence a more careful selection of patients for intensive care might be needed.

At Abdurafi health center, patients with a score of ≥+1 had the highest risk of death (10.4–85.7%) and constituted 26.3% of the case load. Such patients can be triaged towards a unit with the highest level of care or referred to a better established center. They could be admitted in an intensive care unit and treated by experienced VL clinicians. The following investigations could be done routinely: biochemistry (renal and liver function tests etc.), TB screening (chest radiograph, abdominal ultrasound etc.) and HIV monitoring (CD4 counts). Emergency/resuscitation, safest VL treatment (AmBisome)–AmBisome supplies supported by the WHO and other urgent supportive treatment could be provided: oxygen, blood transfusion, broad spectrum antibiotics and nutritional therapy. In VL-HIV co-infected patients, antiretroviral therapy should be initiated as early as possible [6,42].

Patients with a score of 0 had an intermediate risk of death (3.8%) and constituted 21.9% of the case load. Within the scope of ambulatory care and task shifting (treatment by lower cadres of health professionals), such patients could be considered for strategies that include a short stay in a health center or hospital followed by outpatient/decentralized management and task shifting. Patients with a score of –1, had a low risk of death (1.0%), and constituted the majority of the case load (51.8%). This group could be considered in strategies aiming for outpatient/decentralized management and task shifting. Treatment by lower cadres of health professionals could be envisioned. Treatment with SSG could also be safe and appropriate. Nevertheless, more evidence is needed on the impact of the score when applied for such strategies. This score could also be used in clinical research, to standardize patients according to risk groups after inclusion in clinical trials evaluating novel strategies to reduce mortality [53,54].

There are some limitations in the study. As it is retrospective study we could only study predictors from among the variables that we collected. However, our data collection forms were created by VL experts that took into account the main predictors of adverse events in VL patients which are easily documented in our setting. Therefore the majority of the predictors have been studied. Predictors that were not studied (e.g. leucopenia, sepsis etc.) could be integrated in future studies. It is also possible that critical patients may have had more complete data. While there were few missing data in the derivation cohort, this was substantial in the external validation cohort. The majority of the defaulters were never retraced, outcome ascertained, nor were systematic interviews performed to ascertain the reason for defaulting, but a main reason for defaulting in this setting is reported to be an urgent need to return to work and obtain money, and it often occurs while the patient is feeling better. In both cohorts the classification of weakness severity did not follow a recognized standardized grading system, making it difficult to compare our findings with other studies. Lastly, we did not analyze variables such as CD4 counts, WHO stages, antiretroviral therapy that are known to predict death in HIV patients [25]. This is because we aimed to develop a score for all VL patients rather than a specific score for the subgroup of the VL-HIV co-infected. However, this is an important objective for future studies as also outlined in our study protocol in S1 Protocol.

In this study, we developed and externally validated a clinical prognostic score. As simple indicators were used, it is likely to be applicable in most VL treatment settings. While it performed well during external validation, we recommend further validation in other East-African countries, including also regions with a low prevalence of HIV coinfection. Impact studies, assessing whether the use of the score can effectively contribute to reduced mortality–if combined with appropriate treatment strategies–or whether it can make VL treatment programs more (cost)-effective remain to be done as well.

## Supporting information

S1 TRIPOD Checklist TRIPOD checklist: Prediction model development and validation. (DOCX)

S1 Protocol Clinical prognostic tools for mortality in visceral leishmaniasis in a high HIV co-infection burden area in Ethiopia. (PDF)
