# Supplementary material for: Development and external validation of a clinical prognostic score for death in visceral leishmaniasis patients in a high HIV co-infection burden area in Ethiopia
Source: PLoS One. 2017 Jun 5;12(6):e0178996. doi: 10.1371/journal.pone.0178996 (PMC5459471; doi:10.1371/journal.pone.0178996)
Supplement: S1 TRIPOD Checklist — (DOCX) [file pone.0178996.s001.docx]

| **Section/Topic** | **Item** |  | **Checklist Item** | **Page** | **Text extracts** |
| --- | --- | --- | --- | --- | --- |
| **Title and abstract** | | | | |  |
| Title | 1 | D;V | Identify the study as developing and/or validating a multivariable prediction model, the target population, and the outcome to be predicted. | 1 | Development and External Validation of a Clinical Prognostic Score for Death in Visceral Leishmaniasis Patients in a High HIV Co-infection Burden Area in Ethiopia |
| Abstract | 2 | D;V | Provide a summary of objectives, study design, setting, participants, sample size, predictors, outcome, statistical analysis, results, and conclusions. | 2−3 | **Background**  In Ethiopia, case fatality rates among subgroups of visceral leishmaniasis (VL) patients are high.  A clinical prognostic score for death in VL patients could contribute to optimal management and reduction of these case fatality rates. We aimed to identify predictors of death from VL, and to develop and externally validate a clinical prognostic score for death in VL patients, in a high HIV co-infection burden area in Ethiopia.  **Methodology/Principal findings**  We conducted a retrospective cohort study in north west Ethiopia. Predictors with an adjusted likelihood ratio ≥1.5 or ≤0.67 were retained to calculate the predictor score. The derivation cohort consisted of 1686 VL patients treated at an upgraded health center and the external validation cohort consisted of 404 VL patients treated in hospital. There were 99 deaths in the derivation cohort and 53 deaths in the external validation cohort. The predictors of death were: age >40 years (score +1); HIV seropositive (score +1); HIV seronegative (score −1); hemoglobin <6.5 g/dl (score +1); bleeding (score +1); jaundice (score +1); edema (score +1); ascites (score +2) and tuberculosis (score +1). The total predictor score per patient ranged from −1 to +5. A score of −1, indicated a low risk of death (1.0%), a score of 0 an intermediate risk of death (3.8%) and a score of +1 to +5, a high risk of death (10.4−85.7%). The area under the receiver operating characteristic curve was 0.83 (95% confidence interval: 0.79−0.87) in derivation, and 0.78 (95% confidence interval: 0.72−0.83) in external validation.  **Conclusions/Significance**  The overall performance of the score was good. The score can enable the early detection of VL cases at high risk of death, which can inform operational, clinical management guidelines, and VL program management. Implementation of focused strategies could contribute to optimal management and reduction of the case fatality rates. |
| **Introduction** | | | | |  |
| Background and objectives | 3a | D;V | Explain the medical context (including whether diagnostic or prognostic) and rationale for developing or validating the multivariable prediction model, including references to existing models. | 4−5 | In 2014, the VL case fatality rate in Ethiopia was 2.6% [5]. However, differences in case fatality rates among subgroups have been documented. Studies show high case fatality rates among HIV co-infected (7.0−17.4%)[13,14], elderly (12.3%) [15] and malnourished (6.1%) [15] patients.  In East Africa, several predictors of death in VL patients have been identified, such as age, duration of illness, HIV serostatus, spleen size, nutritional status, hemoglobin level, bleeding, jaundice, weakness and tuberculosis (TB) [14–19]. However, no validated clinical prognostic score is currently available to predict death in clinical practice or VL programs. The optimal management and classification of VL severity remains poorly defined, and is highly variable across physicians and treatment sites [12,20,21].  A critical factor that could contribute to the optimal management and reduction of case fatality rates is the availability of evidence-based clinical prognostic tools [22]. Such tools are increasingly used in stratified or risk-based medicine, to identify the individuals requiring close observation and additional testing or treatment [23,24]. On the other hand, those with an excellent prognosis might be treated in an ambulatory way or at a decentralized level [23,24]. For instance, clinical prognostic tools or prediction scores relying on easy to measure clinical and laboratory information have been developed to predict death or morbidity in HIV infected patients [25]. |
|  | 3b | D;V | Specify the objectives, including whether the study describes the development or validation of the model or both. | 5 | In this study, we aimed to identify predictors of death from VL, and to develop and externally validate a clinical prognostic score for death in VL patients in a high HIV co-infection burden area in Ethiopia. |
| **Methods** | | | | |  |
| Source of data | 4a | D;V | Describe the study design or source of data (e.g., randomized trial, cohort, or registry data), separately for the development and validation data sets, if applicable. | 6 | We conducted a retrospective cohort study using routine program data collected using standardized data collection forms. |
|  | 4b | D;V | Specify the key study dates, including start of accrual; end of accrual; and, if applicable, end of follow-up. | 6 | To develop the score, we included all patients diagnosed with VL in Abdurafi health center between January 2008 and December 2013.  To externally validate the score, we included all patients diagnosed with VL at the LRTC between January 2011 and December 2012. |
| Participants | 5a | D;V | Specify key elements of the study setting (e.g., primary care, secondary care, general population) including number and location of centres. | 5−6 | The study was conducted in the Amhara region, north west Ethiopia. Development of the score was conducted at the Abdurafi health center supported by Médecins Sans Frontières (MSF). The health center is located in Abdurafi town, West-Armacheo district − a poor and remote district, with poor access to health care. The health center is upgraded, with a 100 bed capacity, emergency services (such as blood transfusion, oxygen therapy etc.) and capacity to treat VL and HIV co-infected patients. External validation of the score was conducted at the Leishmania Research and Treatment Center (LRTC) at the University of Gondar Hospital supported by the Drugs for Neglected Diseases Initiative (DND*i*). The LRTC is located in Gondar city and is the main referral facility for critically ill or complicated VL cases. The main focus for both treatment centers, is the clinical management of VL and concomitant infections. They are the main VL treatment sites in the high HIV co-infection burden area and medical services are free of charge. |
|  | 5b | D;V | Describe eligibility criteria for participants. | 6 | To develop the score, we included all patients diagnosed with VL in Abdurafi health center between January 2008 and December 2013, whose outcome was cure or in-health center death. We excluded patients if their outcome was transferred-out, defaulted or not reported. To externally validate the score, we included all patients diagnosed with VL at the LRTC between January 2011 and December 2012, whose outcome was cure or in-hospital death. We excluded patients if their outcome was defaulted, treatment failure or not reported. |
|  | 5c | D;V | Give details of treatments received, if relevant. | 7−8 | **Visceral Leishmaniasis treatment**  VL treatment was only administered during admission at the health facility.  **Derivation cohort.** VL disease severity was classified into severe and non-severe, according to MSF guidelines. This classification was based on an algorithm that combined different risk factors of death (weakness, age, body mass index and hemoglobin level)[16,20]. In 2008 to 2012, patients with non-severe primary VL were treated with SSG (Albert David Ltd., Kolkata) at dosages of 20 mg/kg/day (minimum daily dose 200 mg, no maximum dose) by intramuscular injection for a total duration of 30 days. In 2013, the treatment protocol was changed in line with the national guidelines, to the combination of SSG and PM (Gland Pharma Ltd., Hyderabad, India) at dosages of 20 mg/kg/day and 15 mg sulphate/kg/day (11 mg/base/kg/day) respectively by intramuscular injection for a total duration of 17 days [28]. Those with severe primary VL, relapse VL and VL-HIV coinfection were treated with liposomal amphotericin B (AmBisome, Gilead Sciences) at a total dose of 30 mg/kg divided into 6 infusions of 5 mg/kg on alternate days. In 2011, the first line treatment for VL-HIV was changed to a combination therapy of AmBisome at the above dosage and miltefosine (Impavido, Paladin Labs, Montreal, Canada) administered orally for 28 days (100 mg/day in patients weighing more than 25 kg and 50 mg per day in those that were 25 kg or less).  **Validation cohort.** VL disease severity was classified as non-severe and severe, based on clinical judgement [12]. Non-severe primary VL and the majority of severe primary VL, relapse VL and VL-HIV patients received SSG monotherapy at the same dosage as in the derivation cohort. When AmBisome was available, some severe primary VL, relapse VL and VL-HIV patients, received AmBisome monotherapy at the same dosage as in the derivation cohort. |
| Outcome | 6a | D;V | Clearly define the outcome that is predicted by the prediction model, including how and when assessed. | 8 | **Visceral Leishmaniasis treatment outcomes**  Only VL treatment outcomes that occurred during admission at the health facility were documented. There was no patient follow-up after exit from the health facility. In-health center/in-hospital death were defined as death during VL treatment at the health facility. Cure was defined as improvement in symptoms and signs of VL, 17−30 days after treatment initiation (i.e. absence of fever, decrease in spleen size, increase in hemoglobin, weight gain) and a negative parasitological test in VL relapse patients or those with poor treatment response. Transfer-out was defined as referral to another health facility for any reason. Defaulting was defined as absconding from treatment. Treatment failure was defined as a positive parasitological test at the end of treatment. |
|  | 6b | D;V | Report any actions to blind assessment of the outcome to be predicted. | _ | NA |
| Predictors | 7a | D;V | Clearly define all predictors used in developing or validating the multivariable prediction model, including how and when they were measured. | 8−10 | **Data collection and measurement of variables**  From the VL program onset, clinical data were collected using standardized data collection tools and stored in electronic databases. The databases were updated on a daily basis by data managers. The data were collected at admission through history taking, clinical examination, laboratory and/or radiological investigations, and treatment prescriptions (VL regimen).  The following variables were assessed from patient history: age (years), sex, residential status (migrant worker, settler and resident), and the duration of illness (months). While the following were assessed by clinical examination: weight (kilograms), height (meters)/length (centimeters), jaundice, ascites, spleen size (centimeters), bleeding, edema and the level of weakness. Anthropometric parameters were calculated according to WHO guidelines [29–31] [weight-for-length/height z-score in patients 6 months−5 years; body mass index (BMI)-for-age z-score in patients 5−19 years; BMI [weight in kilogram ÷ (height in meter)^2^] in patients >19 years]. In different age groups, severe malnutrition was defined as follows: >19 years (BMI <16.0 kg/m^2^); 5−19 years (BMI-for-age z-score <−3); <5 years (weight-for-length/height z-score <−3). The spleen size (centimeters) was measured from the junction of the anterior axillary line and the left coastal margin to the tip of the spleen. In the derivation cohort, weakness severity was defined according to MSF guidelines [20] as follows: [State of collapse (in adults/older children: unable to sit up unaided and cannot drink unaided. In babies: floppy when held in arms and unable to feed unaided); severely weak (in adults/older children: cannot walk 5 meters without assistance and in babies: unable to sit upright unaided); other types of weakness were classified as “other”]. In the external validation cohort, weakness severity was classified as present or absent, based on clinical judgement.  The following variables were assessed by laboratory and/or radiological investigations. HIV testing was based on MSF and national rapid diagnostic testing algorithms. In Abdurafi Health center, a positive test was defined by two positive serological tests performed in parallel {KHB (Shanghai Kehua Bio-engineering Co-Ltd, Shanghai, China) and STAT-PAK ^TM^ (Chembio HIV1/2, Medford, New York, USA)} and confirmed by the ELISA test {ImmunoComb (Orgenics ImmunoComb® II, HIV 1&2 Combfirm)}. At the LRTC, a positive test was defined by two sequential positive serological tests; KHB followed by STAT-PAK ^TM^ and in case of discrepancy, a tie-breaker test Uni-Gold (Trinity Biotech PLC, Bray, Ireland) was used. TB was diagnosed according to WHO guidelines [32]. Hemoglobin level in grams per deciliter (g/dl) was determined using a hematology analyzer–Beckman Coulter A^c^T diff, Beckman Coulter Inc., 2003, USA. |
|  | 7b | D;V | Report any actions to blind assessment of predictors for the outcome and other predictors. | _ | NA |
| Sample size | 8 | D;V | Explain how the study size was arrived at. | 10 | **Sample size**  To develop the score, we aimed for 10 deaths per variable in the final model. In external validation, the sample size was pre-determined by the available data (53 deaths) [22]. |
| Missing data | 9 | D;V | Describe how missing data were handled (e.g., complete-case analysis, single imputation, multiple imputation) with details of any imputation method. | 11 | To develop the scoring system, the score for each predictor was obtained by calculating the natural logarithm of the adjusted LHR (a value of 0 was allocated to missing data) and rounding this result to the nearest integer. |
| Statistical analysis methods | 10a | D | Describe how predictors were handled in the analyses. | 10 | Other than “age” which was categorized based on information from the literature, continuous variables were dichotomized as guided by receiver operating characteristic curves (ROC), with the optimal cut-off at the point with the highest sum of sensitivity and specificity. The cut-offs were rounded to values that are easy to use in clinical practice. |
|  | 10b | D | Specify type of model, all model-building procedures (including any predictor selection), and method for internal validation. | 10−11 | The score was developed using the Spiegelhalter and Knill-Jones method [33,34].  The score was built as follows: crude likelihood ratios (LHR) were calculated for all predictors and those with a LHR ≥2 or ≤0.5 were selected for use in the next step. To adjust for correlations between predictors, LHR were adjusted using multiple logistic regression. Variables with adjusted LHR ≥1.5 or ≤0.67 were then selected and the model was refitted. This procedure was repeated until all selected variables had an adjusted LHR ≥1.5 or ≤0.67.  Five-fold cross validation was performed. |
|  | 10c | V | For validation, describe how the predictions were calculated. | 10 | The whole data set was used to develop the score. Five-fold cross validation and external validation were performed to evaluate the performance of the score [35]. |
|  | 10d | D;V | Specify all measures used to assess model performance and, if relevant, to compare multiple models. | 11 | The observed probability of death by prognostic score was then calculated. The performance of the score was evaluated by calculating the sensitivity, specificity, positive predictive value (PPV) and negative predictive value (NPV) at different cut−offs. Its overall performance was assessed using the area under the receiver operating characteristic curve (AUROC) and 95% confidence intervals (CI). An AUROC of 0.5 would imply no discrimination, 0.7−<0.8 would imply acceptable discrimination, 0.8−<0.9 would imply excellent discrimination and >0.9 would imply outstanding discrimination [36]. |
|  | 10e | V | Describe any model updating (e.g., recalibration) arising from the validation, if done. | _ | NA |
| Risk groups | 11 | D;V | Provide details on how risk groups were created, if done. | 11 | The observed probability of death by prognostic score was then calculated. |
| Development vs. validation | 12 | V | For validation, identify any differences from the development data in setting, eligibility criteria, outcome, and predictors. | 5−10 | See Items 4, 5, 6 and 7 above |
| **Results** | | | | |  |
| Participants | 13a | D;V | Describe the flow of participants through the study, including the number of participants with and without the outcome and, if applicable, a summary of the follow-up time. A diagram may be helpful. | 12 | **Fig 1. Flow diagram showing the number of patients in the study and their outcomes.** |
|  | 13b | D;V | Describe the characteristics of the participants (basic demographics, clinical features, available predictors), including the number of participants with missing data for predictors and outcome. | 14−17 | **Table 1. Comparison of patient characteristics in the development and external validation cohorts.** |
|  | 13c | V | For validation, show a comparison with the development data of the distribution of important variables (demographics, predictors and outcome). | 14−17 | **Table 1. Comparison of patient characteristics in the development and external validation cohorts.** |
| Model development | 14a | D | Specify the number of participants and outcome events in each analysis. | 14−17  19−21 | **Table 1. Comparison of patient characteristics in the development and external validation cohorts.**  **Table 2. Number of deaths in the derivation cohort, likelihood ratios for predicting death and score by predictor.** |
|  | 14b | D | If done, report the unadjusted association between each candidate predictor and outcome. | 19−21 | **Table 2. Number of deaths in the derivation cohort, likelihood ratios for predicting death and score by predictor.** |
| Model specification | 15a | D | Present the full prediction model to allow predictions for individuals (i.e., all regression coefficients, and model intercept or baseline survival at a given time point). | 19−21 | **Note:** This is the Spiegelhalter and Knill-Jones method. See adjusted likelihood ratios.  **Table 2. Number of deaths in the derivation cohort, likelihood ratios for predicting death and score by predictor.** |
|  | 15b | D | Explain how to the use the prediction model. | 22 | The probability of death ranged from 1.0% for patients with a score of −1 to 85.7% for those with a score of +5. Eight hundred and seventy three patients (51.8%) had a score of −1 and a low risk of death (1.0%), 369 patients (21.9%) had a score of 0 and an intermediate risk of death (3.8%), and 444 patients (26.3%) had a score of +1 to +5 and a high risk of death (10.4−85.7%) (Fig 2).  **Fig 2. Probability of death by clinical prognostic score.** |
| Model performance | 16 | D;V | Report performance measures (with CIs) for the prediction model. | 23 | The AUROC was 0.83 (95% CI 0.79−0.87) in derivation, 0.82 (95% CI 0.77−0.88) in five-fold cross validation and 0.78 (95% CI 0.72−0.83) in external validation (Fig 3).  **Fig 3. ROC curve summarizing the performance of the scoring system during development and external validation.** |
| Model-updating | 17 | V | If done, report the results from any model updating (i.e., model specification, model performance). | _ | NA |
| **Discussion** | | | | |  |
| Limitations | 18 | D;V | Discuss any limitations of the study (such as nonrepresentative sample, few events per predictor, missing data). | 27 | There are some limitations in the study. As it is retrospective study we could only study predictors from among the variables that we collected. However, our data collection forms were created by VL experts that took into account the main predictors of adverse events in VL patients which are easily documented in our setting. Therefore the majority of the predictors have been studied. Predictors that were not studied (e.g. leucopenia, sepsis etc.) could be integrated in future studies. It is also possible that critical patients may have had more complete data. While there were few missing data in the derivation cohort, this was substantial in the external validation cohort. The majority of the defaulters were never retraced, outcome ascertained, nor were systematic interviews performed to ascertain the reason for defaulting, but a main reason for defaulting in this setting is reported to be an urgent need to return to work and obtain money, and it often occurs while the patient is feeling better. Lastly, in both cohorts the classification of weakness severity did not follow a recognized standardized grading system, making it difficult to compare our findings with other studies. Lastly, we did not analyze variables such as CD4 counts, WHO stages, antiretroviral therapy that are known to predict death in HIV patients [25]. This is because we aimed to develop a score for all VL patients rather than a specific score for the subgroup of the VL-HIV co-infected. However, this is an important objective for future studies as also outlined in our study protocol in S2 Protocol. |
| Interpretation | 19a | V | For validation, discuss the results with reference to performance in the development data, and any other validation data. | _ | NA |
|  | 19b | D;V | Give an overall interpretation of the results, considering objectives, limitations, results from similar studies, and other relevant evidence. | 23−24  27  24−25 | Using standardized VL program data from a high VL-HIV burden setting, we identified predictors of death in VL patients, and developed and externally validated a clinical prognostic score for death in VL patients, in a high HIV co-infection burden area in Ethiopia. The overall performance of the score was good with an AUROC of 0.83 (95% CI 0.79−0.87) in derivation and 0.78 (95% CI 0.72−0.83) in external validation. The AUROC is the best measure to assess the overall performance of a clinical score. Positive and negative predictive values also depend on the prevalence of the outcome and hence are context-dependent. In general, an AUROC >0.7 are considered clinically useful and we have used this value to evaluate the performance of our score [36]. For validation, we have used the same value, in addition to the drop in AUROC, relative to the derivation AUROC. The confidence intervals for derivation and validation AUROC overlap, suggesting a minimal drop in diagnostic performance and hence providing rather favorable findings on validation. As most of the predictors are easily identified by health professionals, the tool would be expected to be clinically relevant and easy to use in clinical practice.  Limitations---see above (item 18).  The predictors of death, identified in this study (age >40 years, HIV seropositive, HIV seronegative, hemoglobin <6.5 g/dl, bleeding, jaundice, edema, ascites and TB) are similar to those reported in other studies [6,14–19,39,40]. They indicate the role of VL-HIV co-infection, bone marrow suppression, splenic sequestration and late stage VL disease………………… |
| Implications | 20 | D;V | Discuss the potential clinical use of the model and implications for future research. | 25−27 | The score can enable the early detection of VL cases at high risk of death, which can inform operational, clinical management guidelines and VL program management. Busy treatment programs can use this information to organize patient care according to different patient paths, with different levels of care. However, the decisions on how to use the score, and which cut-offs to apply for decision making require careful consideration as this is context-dependent and largely determined by operational factors. We present the diagnostic performance at different cut-offs, allowing the reader to decide on which cut-off to use in their setting. In relatively better resourced settings (eg. non-governmental organization settings), with sufficient human resources, a higher number of patients could receive closer monitoring/more intensive care. In less resourced settings (eg. overwhelmed public hospitals), applying the same cut-offs might not be feasible, and hence a more careful selection of patients for intensive care might be needed.  At Abdurafi health center, patients with a score of >+1 had the highest risk of death (10.4−85.7%) and constituted 26.3% of the case load. Such patients can be triaged towards a unit with the highest level of care or referred to a better established center. They could be admitted in an intensive care unit and treated by experienced VL clinicians. The following investigations could be done routinely: biochemistry (renal and liver function tests etc.), TB screening (chest radiograph, abdominal ultrasound etc.) and HIV monitoring (CD4 counts). Emergency/resuscitation, safest VL treatment (AmBisome) −AmBisome supplies supported by the WHO and other urgent supportive treatment could be provided: oxygen, blood transfusion, broad spectrum antibiotics and nutritional therapy. In VL-HIV co-infected patients, antiretroviral therapy should be initiated as early as possible [6,42].  Patients with a score of 0 had an intermediate risk of death (3.8%) and constituted 21.9% of the case load. Within the scope of ambulatory care and task shifting (treatment by lower cadres of health professionals), such patients could be considered for strategies that include a short stay in a health center or hospital followed by outpatient/decentralized management and task shifting. Patients with a score of −1, had a low risk of death (1.0%), and constituted the majority of the case load (51.8%). This group could be considered in strategies aiming for outpatient/decentralized management and task shifting. Treatment by lower cadres of health professionals could be envisioned. Treatment with SSG could also be safe and appropriate. Nevertheless, more evidence is needed on the impact of the score when applied for such strategies. This score could also be used in clinical research, to standardize patients according to risk groups after inclusion in clinical trials evaluating novel strategies to reduce mortality [53,54].  In this study, we developed and externally validated a clinical prognostic score. As simple indicators were used, it is likely to be applicable in most VL treatment settings. While it performed well during external validation, we recommend further validation in other East-African countries, including also regions with a low prevalence of HIV coinfection. Impact studies, assessing whether the use of the score can effectively contribute to reduced mortality – if combined with appropriate treatment strategies – or whether it can make VL treatment programs more (cost)-effective remain to be done as well. |
| **Other information** | | | | |  |
| Supplementary information | 21 | D;V | Provide information about the availability of supplementary resources, such as study protocol, Web calculator, and data sets. | 31−32 | The study protocol is in the supplementary information: **S2 Protocol. Clinical prognostic tools for mortality in visceral leishmaniasis in a high HIV co-infection burden area in Ethiopia.**  **Data Availability Statements:**  Requests to access data should be made to the Medical Director of MSF-OCA: Dr. Sid Wong, Medical Director, Médecins Sans Frontières, Operational Centre Amsterdam, Plantage Middenlaan 14, 1018 DD Amsterdam. Email: [sidney.wong@amsterdam.msf.org](mailto:sidney.wong@amsterdam.msf.org). In our context, confidentiality does not only refer to preventing linking data to the identity of an individual patient. Confidentiality also refers to preventing stigmatization of a patient group or community, especially because Médecins Sans Frontières works with very vulnerable communities that may often be stigmatized, excluded, or prosecuted. So even if data are anonymized there are confidentiality issues, and therefore there are ethical reasons to have some restrictions in the process to access data for third parties. Consequently, data will only be made available on a case-by-case basis to bona fide researchers. |
| Funding | 22 | D;V | Give the source of funding and the role of the funders for the present study. |  | CA has received a PhD scholarship granted from the European Union Seventh Framework Program (FP7/2007‐2013) under grant agreement n° 305178 via AfriCoLeish project. The funders had no role in study design, data collection and analysis, decision to publish, or preparation of the manuscript. |

*Items relevant only to the development of a prediction model are denoted by D, items relating solely to a validation of a prediction model are denoted by V, and items relating to both are denoted D;V. We recommend using the TRIPOD Checklist in conjunction with the TRIPOD Explanation and Elaboration document.
