# Supplementary material for: Development and external validation of a clinical prognostic score for death in visceral leishmaniasis patients in a high HIV co-infection burden area in Ethiopia
Source: PLoS One. 2017 Jun 5;12(6):e0178996. doi: 10.1371/journal.pone.0178996 (PMC5459471; doi:10.1371/journal.pone.0178996)
Supplement: S1 Protocol — (PDF) [file pone.0178996.s002.pdf]

# Clinical prognostic tools for mortality in visceral leishmaniasis in a high HIV co-infection burden area in Ethiopia

**<Protocol number (if appl.)>**

**Version 2.0, 19 September 2016**

| | |
|---|---|
| **Sponsor/Commissioner:** | Institute of Tropical Medicine<br>Nationalestraat 155<br>B-2000 Antwerpen - Belgium |
| **Coordinating Investigator:** | Johan Van Griensven |
| **Principal Investigator:** | Acellam Charles Abongomera |

| | |
|---|---|
| **Protocol Number:** | |
| **Title:** | **Clinical prognostic tools for mortality in visceral leishmaniasis in a high HIV co-infection burden area in Ethiopia** |
| **Version:** | Protocol version 2.0 (Dated 19-Sep-2016) |
| **Partnering institutions:** | 1. Institute of Tropical Medicine, Antwerp, Belgium<br>2. Médecins Sans Frontières-Holland, Amsterdam ,The Netherlands<br>3. University of Gondar Hospital, Amhara region, Ethiopia |


| | |
|---|---|
| **Funder:** | AfriCoLeish Project |
| **Sponsor:** | Institute of Tropical Medicine, Antwerp, Belgium |
| **Coordinating Investigator:** | Prof. Dr. Johan Van Griensven |
| **Department:** | Clinical Sciences - Unit of HIV and Neglected Tropical Diseases |
| **Address:** | Institute of Tropical Medicine, Nationalestraat 155, 2000 Antwerp, Belgium |
| **Telephone:** | +32 3 247 64 26 |
| **Fax:** | +32 3 247 64 75 |
| **Email:** | jvangriensven@itg.be |

## TABLE OF CONTENTS

**STATEMENT OF COMPLIANCE & CONFIDENTIALITY** ....4
**LIST OF ABBREVIATIONS** ....6
**SUMMARY** ....7
**1. INTRODUCTION** ....10
1.1 Background ....10
1.2 Rationale ....10
**2. STUDY DESIGN** ....11
**3. STUDY OBJECTIVES** ....11
3.1 Primary Objective ....11
3.2 Secondary Objective ....11
3.3 Tertiary Objective ....11
3.4 The expected outcomes ....11
**4. METHODS** ....12
4.1 Study Setting ....12
4.2 Population ....12
4.3 Sample Size ....12
4.4 Main outcome ....13
4.5 Inclusion Criteria ....13
4.6 Exclusion Criteria ....13
4.7 Data Collection ....13
4.7.1 Study variables ....14
4.7.2 Measurement of variables ....14
4.8 Data Analysis ....17
4.8.1 Quantitative Data Analysis ....17
4.8.2 Score Building ....17
**5. ETHICAL ISSUES** ....18
5.1 Ethical (and Regulatory) Review ....18
5.2 Risks and Benefits ....18
5.3 Investigators ....18
5.4 Facilities ....19
5.5 Confidentiality and privacy ....19
5.6 Funding ....19
**6. MONITORING AND QUALITY CONTROL** ....19
6.1 Human resources and responsibilities ....19
**7. TIME SCHEDULE** ....19
**8. DATA MANAGEMENT AND ARCHIVING** ....20
8.1 Data Management ....20
8.2 Archiving ....20
**9. DISSEMINATION OF RESULTS** ....20
**10. REFERENCES** ....22
**11. ANNEXES** ....24
Annex 1: kala azar treatment card ....24

## STATEMENT OF COMPLIANCE & CONFIDENTIALITY

The information contained in this study protocol is privileged and confidential. As such, it may not be disclosed unless specific permission is given in writing by the ITM or when such disclosure is required by federal or other laws or regulations. These restrictions on disclosure will apply equally to all future information supplied which is privileged or confidential.

Once the final protocol has been issued and signed by the Investigator(s) and the authorized signatories, it cannot be informally altered. Protocol amendments have the same legal status and must pass through the mandatory steps of review and approval before being implemented.

By signing this document, the Investigator commits to carry out the study in compliance with the protocol, the applicable ethical guidelines like the Declaration of Helsinki and consistent with international scientific standards as well as all applicable regulatory requirements. The Investigator will also make every reasonable effort to complete the study within the timelines designated.

**PRINCIPAL INVESTIGATOR:**

Dr. Acellam Charles Abongomera Date: 19-September-2016

Signed:

**COORDINATING INVESTIGATOR:**

Prof. Dr. Johan Van Griensven Date: 19-September-2016

Signed:

**Co-Investigator Médecins sans Frontières Holland**

Dr. Koert Ritmeijer

**Co-Investigator Ethiopia**

Dr. Ermias Diro

**Other collaborators**

| | |
|---|---|
| Prof. Dr Lut Lynen | ITM, Antwerp (Clinical Sciences Department) |
| Dr. Jozefien Buyze | ITM, Antwerp (Clinical Sciences Department) |
| Dr. Henok  Admassu | Médecins Sans Frontières Holland |
| Dr. Elshafie Mohamed Ahmed | Médecins Sans Frontières Holland |

## LIST OF ABBREVIATIONS

| | |
|---|---|
| ART | Antiretroviral Therapy |
| BMI | Body mass index |
| DAT | Direct agglutination test |
| DND*i* | Drugs for Neglected Diseases Initiative |
| HIV | Human immunodeficiency virus |
| LHR | Likelihood ratio |
| LRTC | Leishmania Research and Treatment Centre |
| ITM | Institute of Tropical Medicine, Antwerp, Belgium |
| IRB | Institutional Review Board |
| MSFH | Médecins Sans Frontières Holland |
| PhD | Doctor of Philosophy |
| PI | Principal Investigator |
| ROC | Receiver operating characteristic curve |
| TB | Tuberculosis |
| VL | Visceral Leishmaniasis |

## SUMMARY

**Title:** Clinical prognostic tools for mortality in visceral leishmaniasis in a high HIV co-infection burden area in Ethiopia

### Primary objective

To develop a mortality prognostic score for visceral Leishmaniasis (VL) patients with an area under the receiver operating characteristic curve (ROC) of more than 0.75.

### Secondary objective

1. To determine the predictive factors for mortality in VL patients.

2. To determine the predictive factors for mortality in VL-HIV co-infected patients.

### Tertiary Objective

To develop a mortality prognostic score for VL-HIV co-infected patients with an area under the receiver operating characteristic curve (ROC) of more than 0.75.

### Study design

A retrospective cohort analysis using prospectively collected standardized program data.

### Study setting

Development of the tool: Abdurafi health centre in Abdurafi (Ethiopia) supported by Médecins Sans Frontières Holland.

External validation of the tool: Leishmania Research and Treatment Center at the University of Gondar Hospital supported by the Drugs for Neglected Diseases Initiative.

### Study population

Development of the tool: All patients diagnosed with primary and relapse VL between January 2008 to December 2013.

External validation of the tool: All patients diagnosed with primary and relapse VL between January 2011 to December 2012.

### Sample Size

1. Development of the score:

   All VL patients: VL patients diagnosed between January 2008 to December 2013. There were 1729 patients diagnosed in this period among whom 99 died.

   VL-HIV co-infected patients. VL patients diagnosed between January 2008 to December 2013. There were 345 patients diagnosed in this period among whom 51 died.

2. External validation:

<u>All VL patients</u>: VL patients diagnosed between January 2011 to December 2012. There were 526 patients diagnosed in this period among whom 53 died.

<u>VL-HIV co-infected patients.</u> VL patients diagnosed between January 2011 to December 2012. There were 55 patients diagnosed in this period among whom 28 died.

**Main outcome**

In health-centre/hospital mortality

**Inclusion criteria**

1. Cured patients 2. Dead Patients

**Exclusion criteria**

1. Transferred patients 2. Defaulted patients

The transferred and defaulted patients were few (approximately 4%). Additionally, patients with treatment failure were excluded from the external validation cohort. There were no patients with treatment failure in the derivation cohort.

**Study variables**

The following variables will be collected and analyzed. They are identified by literature review for predictive factors for mortality and consideration of routinely collected program data

- Demographics: age, sex
- Clinical
    - On admission:
        - Duration of illness
        - Presence or absence of weakness, ascites, peripheral edema, bleeding, jaundice, tuberculosis
        - Spleen size
        - Body mass Index (BMI)
        - Hemoglobin
- VL history (Primary VL versus VL relapse); VL treatment received
- HIV status, Antiretroviral use, $CD4^+$ T lymphocyte count

**Statistical methods**

Statistical analysis will be done with Stata version 13.1. The main exposure variables in 10% of the patient charts will be validated against the records in the excel database. Univariate and multivariate logistic regression analysis will be done. The spiegelhalter and Knill Jones method will be used to develop the score. Predictors with an adjusted likelihood ratio of $\leq$0.67 and $\geq$1.5 will be used to develop the score. A receiver operating characteristic curve will be constructed and cut off selected with the Zweig and Campbell formula.

**Main results**

1. The predictive factors for mortality in VL patients.

2. The predictive factors for mortality in VL-HIV co-infected patients.

3. The performance of the prognostic score in VL patients as per receiver operating characteristic curve with sensitivity, specificity, predictive values and likelihood ratios at different cut-offs of the score.

4. The performance of the prognostic score in VL-HIV co-infected patients as per receiver operating characteristic curve and with sensitivity, specificity, predictive values at different cut-offs of the score.

5. A clinical algorithm identifying groups of VL patients with varying risk of mortality.

6. A clinical algorithm identifying groups of VL-HIV co-infected patients with varying risk of mortality.

**Possible limitations**

1. The clinical records of critical patients may contain more complete data compared to the rest of the patients thus creating some bias.

2. There will be some missing data however they are few (<1%). Médecins Sans Frontières Holland has multi-country standardized data collection tools (See Annexe 1). Standardized data collection forms were used to collect the data at the Leishmania Research and Treatment Centre.

3. The definition of the variable "weakness" is not based on the Karnofsky performance scale Index or standardised system at both VL treatment sites.

**Impact**

Once validated it can help identify patients at high risk of mortality, who might benefit more from the more expensive and less accessible AmBisome; and from intensive monitoring by the scarce human resources; it can help in the decentralization of care by deciding which patients may be treated in the hospital and which ones treated in the health centre and lastly, it may play a role in selection of patients to target groups in future studies.

# 1. INTRODUCTION

## 1.1 Background

Visceral Leishmaniasis (VL) or Kala-azar is a disseminated protozoan infection caused by *Leishmania donovani* species complex and transmitted through the bite of the female sandfly.[1] The anthroponotic form is caused by *L. donovani* and is the most prevalent. It occurs in East Africa, Bangladesh, India and Nepal. The zoonotic form with dogs as the main reservoir is caused by *L. infantum* and occurs in the Mediterranean basin, China, the Middle East and South America. [2,3] VL is present in more than 70 countries and 200 million people are at risk. Annually there are 500,000 new cases and 50,000 deaths. 90% of VL cases occur in only 6 countries namely Bangladesh, Brazil, Ethiopia, India, Nepal and Sudan. In Ethiopia, 2000-4500 VL cases occur annually. [4–6]

Human immunodeficiency virus (HIV) is one of the main risk factors for VL[7,8] and consequently the HIV epidemic has significantly increased VL caseloads especially in East Africa. In Ethiopia, HIV prevalence among VL patients is 20-40%, the highest co-infection rate in the world.[4,5] VL and HIV are both immunosuppressive and act synergistically with detrimental effects.[9,10] Co-infected patients show atypical presentation, have several VL relapses, higher rates of drug toxicity, treatment failure and mortality.[11–14] The high treatment failure rates are a risk factor for the development of drug resistance.[15,16]

VL is fatal without treatment and the main drugs used for treatment are antimonials and Liposomal Amphotericin B (AmBisome). Sodium Stibogluconate is a commonly used antimonial and a full course of treatment costs 56 US dollars while a course of AmBisome is 13 times more expensive.[17] Antimonials are more toxic than AmBisome especially in HIV co-infected patients, the mortality rate among VL-HIV co-infected patients on antimonial therapy varies from 6.8-33%. This is 4-10 times higher than in non HIV infected patients.[11–14]. However their favourable cost makes them the first choice of treatment in resource limited settings. Although antiretroviral treatment (ART) has been gradually introduced in Ethiopia as well, the epidemiological and clinical impact on VL-HIV co-infection has been poorly documented.

## 1.2 Rationale

The optimal management of severe cases remains poorly identified and is highly variable across physicians and treatment sites. A critical factor that could contribute in reducing adverse outcomes include the availability of evidence-based prognostic tools. Such tools are increasingly used in stratified or risk-based medicine, to identify the individuals requiring close observation, and additional testing or treatment. On the other hand, individuals with an excellent prognosis might be treated in an ambulatory way or at the decentralized level.

To be clinically relevant and influence clinical care or VL programs, identified prognostic factors should be integrated in easy to use tools. For instance, clinical prognostic tools or prediction scores have been developed to predict mortality or morbidity in HIV infected patients, relying on easy to measure clinical and laboratory information.[18] While a scoring system for VL mortality has been developed in Sudan, it has never been validated.[19] Moreover, it was developed over ten years ago within a specific setting in South Sudan; it is however not known to what extent it is applicable to the Ethiopian setting, with high HIV co-infection rates.

Using standardized VL program data from a high VL-HIV burden setting, we aim to identify independent risk factors associated with death in VL patients and **to develop and externally validate a clinical prediction score for VL mortality**. Such a tool could contribute to reducing

VL case fatality by allowing the early identification of patients at higher risk for death from VL, and to implement focused strategies. A validated clinical prediction score could help identify patients at high risk of mortality, who might benefit more from the scarcely available AmBisome, intensive monitoring by the scarce human resources; it can help in the decentralization of care by deciding which patients may be treated in the hospital and which ones treated in the health centre and lastly, it may play a role in selection of patients to target groups in future studies. Clinical prediction scores are potentially highly useful tools but their study for use in resource limited settings is very limited. Our studies will be one of the few dedicated to this.

## 2. Study Design

This is a retrospective cohort study using prospectively collected standardized program data. It is foreseen to last for 7 months.

## 3. Study Objectives

### 3.1 Primary Objective

To develop a mortality prognostic score for VL patients with an area under the receiver operating characteristic curve (ROC) of more than 0.75.

### 3.2 Secondary Objective

1. To determine the predictive factors for mortality in VL patients.
2. To determine the predictive factors for mortality in VL-HIV co-infected patients.

### 3.3 Tertiary Objective

To develop a mortality prognostic score for VL-HIV co-infected patients with an area under the receiver operating characteristic curve (ROC) of more than 0.75.

### 3.4 The expected outcomes

1. The predictive factors for mortality in VL patients.
2. The predictive factors for mortality in VL-HIV co-infected patients.
3. The performance of the prognostic score in VL patients as per ROC and with sensitivity, specificity, predictive values at different cut-offs of the score.
4. The performance of the prognostic score in VL-HIV co-infected patients as per ROC and with sensitivity, specificity, predictive values at different cut-offs of the score.
5. A clinical algorithm identifying groups of VL patients with varying risk of mortality.
6. A clinical algorithm identifying groups of VL-HIV co-infected patients with varying risk of mortality.

# 4. METHODS

## 4.1 Study Setting

**Development of the tool** will be conducted in Abdurafi Health centre in Abdurafi town, West-Armacheo district, North West Ethiopia. It is a poor and remote district with poor access to health. It has one of the highest burden of VL and HIV in Ethiopia. Seasonal crop farming is one of the main activities and approximately 300,000 migrant workers travel in and out of the region seasonally. Commercial sex work is also very common.

The health centre belongs to the Ethiopian bureau of health and has been supported by Médecins Sans Frontières Holland (MSFH) since 2004. It focusses on the clinical management of VL, HIV and concomitant infections. It is one of the main VL treatment sites in Ethiopia and medical services are free of charge. The majority of the patients treated are migrant workers.

We consider this site suitable for our study because of the following facts:

(1) Ethiopia is one of the top six countries in the world with the highest burden of VL and it has the highest burden of VL-HIV Co-infection.

(2) Readily available high quality data: Since the VL program onset in 2004, standardized data collection sheets (patient cards) have been used, from which data are transferred to an electronic database. (See Annex 1) Clinical teams are trained on standardized clinical procedures and data collection and a data quality system is in place.

(3) The VL mortality rate remains high in the health center.

**External validation of the tool** will be conducted at the Leishmania Research and Treatment Center (LRTC) at the University of Gondar Hospital supported by the Drugs for Neglected Diseases Initiative (DND*i*). The LRTC is located in Gondar city and is the main referral facility for critically ill or complicated VL cases. The main focus for this centre, is the clinical management of VL and concomitant infections and clinical research on VL. It is one of the main VL treatment sites in the high HIV co-infection burden area and medical services are free of charge. We consider this site suitable because along with Abdurafi health centre they are the main VL treatment sites in the high HIV co-infection burden area. Furthermore there is readily available high quality data collected using standardized data collection forms.

## 4.2 Population

**Development of the tool:** All patients diagnosed with primary and relapse VL between January 2008 to December 2013.

**External validation of the tool:** All patients diagnosed with primary and relapse VL between January 2011 and December 2012

## 4.3 Sample Size

**Development of the tool:**

**All VL patients:** The score will be developed on the 1729 patients diagnosed between January 2008 to December 2013, among whom 99 died. According to a rule of thumb, ten deaths are required for each parameter in the logistic regression model.[20] Therefore the final model should

contain no more than 9 parameters. Five-fold cross validation will be performed to evaluate the performance of the score.

**VL-HIV co-infected patients.** VL Patients diagnosed between January 2008 to December 2013. There were 345 patients diagnosed in this period among whom 51 died. Therefore the final model should contain no more than 5 parameters. Five-fold cross validation will be performed to evaluate the performance of the score.

**External validation of the tool**:

**All VL patients:** The sample size will be pre-determined by the available data (53 deaths).[26]

**VL-HIV co-infected patients:** The sample size will be pre-determined by the available data (28 deaths)

### 4.4 Main outcome

The main outcome measured is mortality during VL treatment (In-health centre/hospital mortality).

### 4.5 Inclusion Criteria

In the derivation and external validation cohort, we will include all patients with the outcomes mentioned below.

1. Cured
2. Dead

### 4.6 Exclusion Criteria

In the derivation and external validation cohort, we will exclude all patients with the outcomes mentioned below.

1. Transferred
2. Defaulted

The transferred and defaulted patients were few (approximately 4%). Additionally in the external validation cohort, we will exclude all patients with treatment failure. There were no patients with treatment failure in the derivation cohort.

### 4.7 Data Collection

The study variables are extracted from an excel data base that contains the clinical records of VL patients. The main exposure variables in 10% of the patient charts will be validated against the records in the excel database.

### 4.7.1 Study variables

The main exposure variables mentioned below where identified by literature review for predictive factors for mortality[13,19,21–23]and taking into consideration the data routinely collected in the MSFH program.

- Demographics: age (years) , sex (male or female)
- Clinical
  - On admission:
    - Duration of illness (weeks or months)
    - Presence or absence of weakness, ascites, peripheral edema, bleeding, jaundice, tuberculosis (TB)
    - Spleen size (cm)
    - Body mass Index (BMI) (in $kg/m^2$)
    - Hemoglobin (g/dl)
- VL history (Primary VL versus VL relapse); VL treatment received (Antimonial based or other)
- HIV status (negative, positive, discordant)
- Antiretroviral (ART) use (yes or no; duration)
- $CD4^+$ T lymphocyte count (cells/µl), most recent result

### 4.7.2 Measurement of variables

- **HIV status**

HIV testing is done routinely in all VL suspects upon admission, based on rapid diagnostic testing algorithms according to MSFH and national guidelines.

- **VL history (Primary versus Relapse)**

VL is diagnosed according to MSFH and national guidelines.[24] A suspected VL patient is one who presents with prolonged fever (2 weeks or more) with splenomegaly and/or lymphadenopathy and/or wasting AND has lived or travelled to a known visceral leishmaniasis endemic area. Suspected VL patients undergo further diagnostic evaluations.

A primary VL case is defined as a patient that has never been treated for VL and has a positive rK39 rapid diagnostic test (DiaMed-IT-Leish) or a high-titer (≥1:3200) of a direct agglutination test (DAT) or a borderline DAT titre (1:800–1:1600) and a positive parasitological test (spleen, bone marrow or Lymph node). Aspirates of the spleen, bone marrow or lymph node are stained with Giemsa and examined under a microscope. At the LRTC, DAT is not performed, rK39

negative patients undergo tissue aspiration (spleen, bone marrow or lymph node) and VL is confirmed parasitologically.

A parasitological test refers to microscopic examination for *Leishmania* amastigotes in giemsa stains of tissue aspirates (spleen, bone marrow or Lymph node). The results are logarithmically graded from 0 (0 parasites/1000 fields) to 6 (>100 parasites/field).

A relapse VL case is defined as a patient previously treated for VL with a positive parasitological test (spleen, bone marrow or Lymph node).

Suspected VL patients with negative serological tests or also negative parasitological tests are evaluated for possible differential diagnoses. If no differential diagnoses are found the patients are rescreened for VL one to two weeks later.

**Primary VL diagnostic algorithm in Abdurafi Health centre**

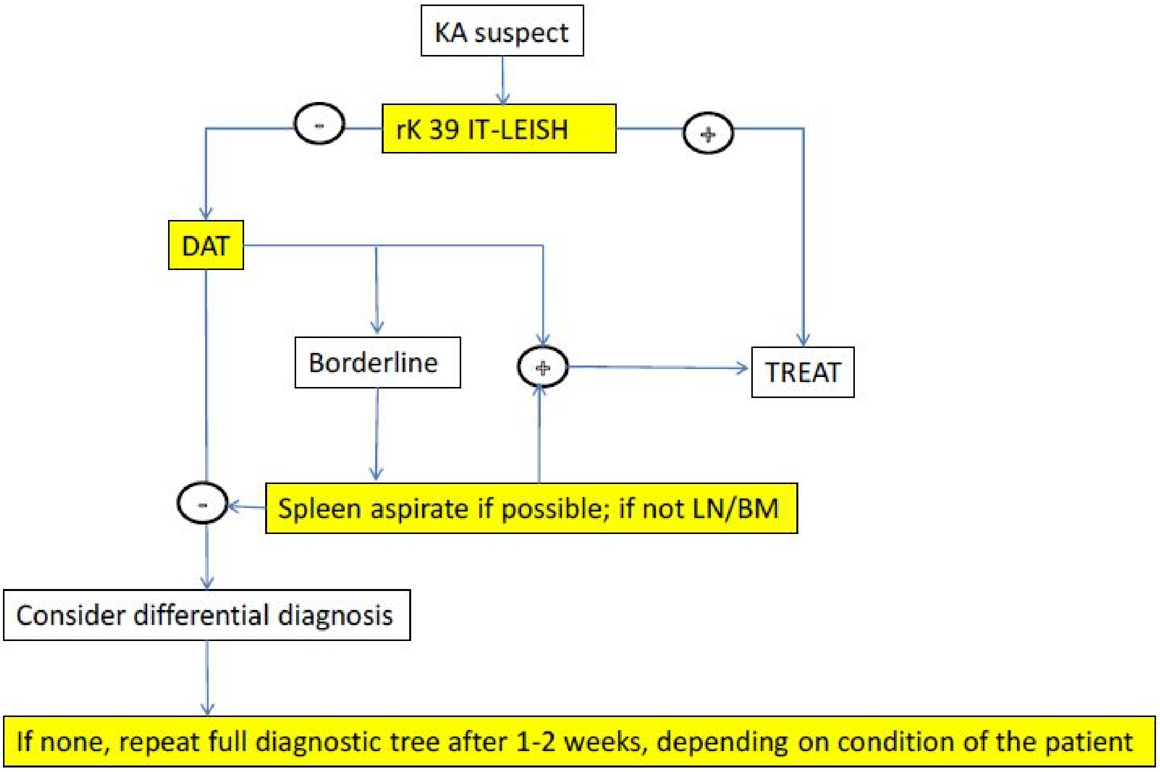


- **$CD4^+$ T lymphocyte count**: This is measured in cells/µl using FACS counter (BD FACS Calibur flow cytometer, 2009, USA).
- **ART use:** This is defined from the patient's history and/or records from the HIV clinic and/or visual inspection of the drugs.
- **Body Mass Index:** The BMI is calculated with the following formula.

$$BMI = \frac{\text{Weight (in Kg)}}{\{\text{Height (in m)}\}^2}$$

The weight is measured with a mechanic scale to the nearest 0.5 Kg.
The height is measured with a stadio meter in centimeters to the nearest 0.5 cm.

- **Jaundice:** This is defined by clinical examination revealing a yellowish coloration of the sclera and reported as either present or absent.

- **Duration of illness:** This is defined from the patient's history and reported in weeks or months.

- **Tuberculosis:** This is defined as follows.

- Absence of TB: Absence of history and signs suggestive of TB suspect or a suspected TB patient with negative sputum smear results and/or normal radiograph findings and/or normal abdominal ultrasound findings.

- Presence of TB: History and signs suggestive of TB suspect with positive sputum smear results and/or suggestive radiograph findings and/or suggestive abdominal ultrasound findings or diagnosed by a TB expert.

- **Age**: This is defined from the patient's history and measured in years.

- **Haemoglobin:** The haematological analysis is done by a haematology analyser – Beckman Coulter A$^{c}$T diff, Beckman Coulter Inc., 2003, USA. Haemoglobin is measured in g/dl routinely upon admission.

- **Severe Weakness:** The MSFH operational definitions of "Severe Weakness" are as defined below;

- State of collapse = score 5

Definition of collapse in adults/older children: unable to sit up unaided AND cannot drink unaided.

Definition of collapse in babies: floppy when held in arms AND unable to feed unaided.

- Severely weak = score 3

Definition of severe weakness in adults/older children: cannot walk 5 m without assistance.

Definition of severe weakness in babies: unable to sit upright unaided.

- Other types of weakness = score 0

At the LRTC, the definition of weakness was based on the clinician's assessment and classified as either present or absent.

- **Ascites:** This is defined by clinical examination and reported as either present or absent.

- **Spleen size at admission:** This is measured in the anterior axillary line from the coastal margin to the tip of the spleen. The measurements are recorded in centimetres.

- **Bleeding:** This is defined by clinical examination and reported as either present or absent.

- **Oedema:** This is defined by clinical examination and reported as either present or

absent.

- **Treatments:** This is the VL treatment regimen used during treatment and will be measured in 2 groups. A treatment regimen containing an antimonial and a treatment regimen without an antimonial.

  **Definitions of outcome variables:**

- **Death:** Death during VL treatment at the health center.

- **Cure:** Improvement in symptoms and signs of VL 20-30 days after VL treatment initiation i.e. absence of fever, decrease in spleen size, increase in haemoglobin level, weight gain and a negative test of cure (parasitological test) where indicated.

- **Transfer:** A VL patient on treatment that is sent to a higher level health facility for better management or sent to another health facility for compassionate reasons e.g. proximity of that health facility to the patients' home.

- **Defaulter:** A VL patient on treatment that absconds from treatment and does not return or returns but the VL treatment has to be restarted from the first day.

- **Treatment failure:** It is defined as a positive parasitological test at the end of VL treatment.

### 4.8 Data Analysis

#### 4.8.1 Quantitative Data Analysis

Quantitative data analysis will be done and will consist of univariate and multivariate logistic regression analysis. Stata version 13.1 will be used for this analysis. The ITM Principal Investigator (PI) and statistician will be responsible for carrying out the data analysis.

The predictors for mortality that will be analyzed are indicated under section 4.7.1. The main outcome measured is in-health center/hospital mortality. The aim of the analysis is to identify independent predictors for mortality. These independent predictors of mortality shall be used to develop a clinical prediction score as explained below under section 4.8.2.

#### 4.8.2 Score Building

The score will be developed using the Spiegelhalter and Knill-Jones method.[25] Derivation of the clinical prediction score will be done in the 2008-2013 data. Five-fold cross validation and external validation will be performed [26]. The procedure of building the score is as outlined below.

- Crude likelihood ratios (LHR) will be calculated for all variables and those with a LHR ≥2 or ≤0.5 will be selected for inclusion in the score building.

- To adjust for correlations between predictors, LHR will be adjusted using multivariate logistic regression.

- Variables independently associated with mortality with a LHR ≥1.5 or ≤0.67 will be retained.

- To develop the scoring system, the predictor score for each predictor will be obtained by calculating the natural logarithm of the adjusted LHR and rounding this result to the nearest integer.

- Summing the predictor scores of the individual's risk factors will yield the total predictor score for each patient.

- The performance of the clinical prediction score using the validation dataset will be evaluated by calculating the sensitivity, specificity, positive predictive value, negative predictive value, LHR at different cut offs; it's overall performance will be assessed using the area under the receiver operating characteristic curve and 95% CI.

- Based on these data, different risk groups can be identified using different cut-offs (e.g. low, intermediate and high risk groups).

## 5. ETHICAL ISSUES

### 5.1 Ethical (and Regulatory) Review

This protocol and any subsequent amendments will be submitted for approval to the Institutional Review Board of the ITM, the MSFH Ethics Committee and the Ethical Review Board of the Institute of Public Health, Gondar University. The study will be carried out according to the principles stated in the Declaration of Helsinki, all applicable regulations and according to established international scientific standards. Ethical approval for use of retrospective data from LRTC was obtained from University of Gondar IRB.

### 5.2 Risks and Benefits

This study is observational and not experimental. It is consists of retrospective analysis of anonymized routinely collected program data. There is no direct risk for the patients. The final results of the study will be beneficial for better clinical management of patients. All patients are treated following the national, MSFH guidelines and latest scientific evidences.

### 5.3 Investigators

The principal investigator of this study at the ITM is Dr. Acellam Charles Abongomera. He is a medical doctor who was involved in the clinical care of VL patients in Abdurafi health center from November 2013 till March 2015. He is currently a Pre-PhD student at ITM and is responsible for the development and smooth running of the study protocol. He has participated to a short course in clinical research and evidence based medicine (SCREM) at the ITM Antwerp in 2014 (April-June) where the development of this protocol was started. While working for MSFH in Abdurafi, he already invested quite some time in further cleaning and validation of the available databases. Additional queries relating to the data will be solved via Charles and Koert.

The coordinating investigator for this research at the ITM is Prof. Dr Johan Van Griensven he is the head of the HIV and Neglected Tropical diseases unit at the clinical sciences department of the ITM, researcher, ITM member of the AfriCoLeish project and has done extensive work on VL and VL-HIV coinfection.

Dr. Koert Ritmeijer will be the key co-investigator for MSFH. He has been extensively involved in diagnostic, treatment and operational studies on VL and VL-HIV co-infection. He is also very

well connected within the VL scientific and programmatic networks, including several research consortia. He is responsible for the smooth running of the protocol at the MSFH site.

Dr. Ermias Diro will be a key co-investigator in Ethiopia. He is extensively involved in clinical studies on VL and VL-HIV co-infection and also conducted his PhD (Clinical management of visceral leishmaniasis in HIV patients in Ethiopia) via ITM.

### 5.4 Facilities

Abdurafi is a well-functioning health center supported by MSFH with a main focus on VL clinical management. It is part of the AfriCoLeish Collaboration with a lot of experience in the management of VL. The MSFH supported health centre has 100 beds, its own laboratory and is well staffed. Clinical teams are trained on standardized clinical procedures and data collection by experts from MSFH, Gondar Hospital and ITM. The medical consultations are supervised by an MSFH physician experienced in VL case management and supported by the MSFH VL advisor. A data manager trained by MSFH is responsible for entering the data into a computerized database. The data entry will be monitored by the ITM PI and MSFH co-investigator.

The LRTC, located in Gondar University Hospital is supported by the DND*i* and has 24 beds, its own laboratory and is adequately staffed. Several clinical trials are conducted at the centre. ITM also has an institutional agreement with Gondar University.

### 5.5 Confidentiality and privacy

The VL database contains routine program data that are not collected for the main aim of research. Patient's records in the databases are anonymized. The data entry will be in an anonymous way. Patients will not be traced and confidentiality agreements will be respected in case of review of patient's records containing identifiers e.g. patient medical charts and laboratory reports by the PI.

### 5.6 Funding

The PI has a scholarship via the FP7 AfriCoLeish project. Any additional costs for the conduct of the study will be covered via the same grant.

## 6. MONITORING AND QUALITY CONTROL

In case ITM would be willing to carry out monitoring activities on site, the Abdurafi health center and the LRTC, will allow the ITM's monitor or a monitor designated by the ITM access to source documents or the data bases present at the health center.

### 6.1 Human resources and responsibilities

The principal investigator of the Study at the ITM is Dr. Acellam Charles Abongomera. Prof. Dr Johan Van Griensven is the coordinating investigator at the ITM. Dr. Koert Ritmeijer is key co-investigator for MSFH. Dr. Ermias Diro is a key co-investigator in Ethiopia. The responsible for the development of the study protocol is Dr. Acellam Charles Abongomera. The statistical analysis will be done by Dr. Jozefien Buyze, a scientific staff of the Clinical trials unit at the ITM. He is a lecturer in statistics at the ITM.

## 7. TIME SCHEDULE

<u>September 2016:</u>

- Presentation of the protocol to the ITM IRB.

October 2016:

- Data analysis

November 2016 - April 2017:

Two Publications submitted

- First publication will be submitted in November 2016 (Development and External Validation of a Clinical Prognostic Score for Death in Visceral Leishmaniasis Patients in a High HIV Co-infection Burden Area in Ethiopia)

- Second publication in April 2017 (Development and External Validation of a Clinical Prognostic Score for Death in Visceral Leishmaniasis and HIV Co-infected patients in Ethiopia)

## 8. DATA MANAGEMENT AND ARCHIVING

### 8.1 Data Management

All the patient files (source documents) are available in a locked cabinet at the health center and LRTC. The patients' clinical records are anonymized and stored in electronic data bases. The VL data base is stored in Excel sheet 2010 format while the HIV data base is in Microsoft Access office 2010 format.

The study variables were extracted and encoded in Excel 2013 format. We validated the records in 10% of the patient charts against the records in the excel data tool during encoding in the excel 2013 format database. The duration on ART and CD4 counts were extracted from the HIV data base and validated as described above. Double data entry and querying was done by the PI and data manager. Procedures for source data verification are in place. All data entry was anonymized and the data shall be kept in a computer that is password protected and only accessible to the PI and data manager.

### 8.2 Archiving

The main source of data that will be used for the score development are the available electronic databases. The original and final databases will be locked and kept by the PI for a minimum of five years. All the patient file/cards (source documents) are available at the site, they are anonymised, placed in binders in a locked cabinet and under the responsibility of MSFH and LRTC respectively. The source documents (patient files) will remain the property and responsibility of MSFH and LRTC respectively.

## 9. DISSEMINATION OF RESULTS

We foresee that the results of this study will be of sufficient medical importance to warrant publication in an international peer-reviewed journal. All research partners will be involved in data analysis, and a joint agreement will be made on the content of the publication and communication strategy. The sponsor will ensure that all those who have significantly been involved in the study (according to ICMJE guidelines) will be included as co-authors in the main publication of the findings. Given his key role in this study and given the fact that this study is part of his PhD, the PI-ITM will be first author for the main study paper reporting on the clinical prognostic tool for mortality in visceral leishmaniasis, under the condition that he continues to

act as PI during the study. This refers to the main paper on the score development and not to possible other publications on other aspects of the study.

## 10. REFERENCES

1. Murray, H. W., Berman, J. D., Davies, C. R. & Saravia, N. G. Advances in leishmaniasis. *Lancet* **366,** 1561–77 (2005).
2. Chappuis, F. *et al.* Visceral leishmaniasis: what are the needs for diagnosis, treatment and control? *Nat. Rev. Microbiol.* **5,** 873–82 (2007).
3. Herwaldt, B. L. Leishmaniasis. *Lancet* **354,** 1191–1199 (1999).
4. Alvar, J. *et al.* The relationship between leishmaniasis and AIDS: the second 10 years. *Clin. Microbiol. Rev.* **21,** 334–59 (2008).
5. Diro, E. *et al.* Visceral Leishmaniasis and HIV coinfection in East Africa. *PLoS Negl. Trop. Dis.* **8,** e2869 (2014).
6. WHO. *Working to overcome the global impact of neglected tropical diseases First WHO report on neglected tropical diseases*. (2010).
7. Gradoni, L., Scalone, A., Gramiccia, M. & Troiani, M. Epidemiological surveillance of leishmaniasis in HIV-1-infected individuals in Italy. *AIDS* **10,** 785–791 (1996).
8. Lopez-Velez, R. *et al.* Clinicoepidemiologic characteristics, prognostic factors, and survival analysis of patients coinfected with human immunodeficiency virus and Leishmania in an area of Madrid, Spain. *Am. J. Trop. Med. Hyg.* **58,** 436–43 (1998).
9. Olivier, M., Badaró, R., Medrano, F. J. & Moreno, J. The pathogenesis of Leishmania/HIV co-infection: cellular and immunological mechanisms. *Ann. Trop. Med. Parasitol.* **97 Suppl 1,** 79–98 (2003).
10. Tremblay, M., Olivier, M. & Bemier, R. Leishmania and the Pathogenesis of HIV Infection. *Parasitol. Today* **12,** 257–61 (1996).
11. Ritmeijer, K. *et al.* Ethiopian visceral leishmaniasis: generic and proprietary sodium stibogluconate are equivalent; HIV co-infected patients have a poor outcome. *Trans. R. Soc. Trop. Med. Hyg.* **95,** 668–672 (2001).
12. Ritmeijer, K. *et al.* Limited effectiveness of high-dose liposomal amphotericin B (AmBisome) for treatment of visceral leishmaniasis in an Ethiopian population with high HIV prevalence. *Clin. Infect. Dis.* **53,** e152-8 (2011).
13. Hurissa, Z. *et al.* Clinical characteristics and treatment outcome of patients with visceral leishmaniasis and HIV co-infection in northwest Ethiopia. *Trop. Med. Int. Health* **15,** 848–55 (2010).
14. Cota, G. F., de Sousa, M. R., Fereguetti, T. O. & Rabello, A. Efficacy of anti-leishmania therapy in visceral leishmaniasis among HIV infected patients: a systematic review with indirect comparison. *PLoS Negl. Trop. Dis.* **7,** e2195 (2013).
15. Diro, E. *et al.* High parasitological failure rate of visceral leishmaniasis to sodium stibogluconate among HIV co-infected adults in Ethiopia. *PLoS Negl. Trop. Dis.* **8,** e2875 (2014).
16. Chakravarty, J. & Sundar, S. Drug Resistance in Leishmaniasis. *J. Glob. Infect. Dis.* **19,** 167–176 (2010).
17. World Health Organization. *Control of the leishmaniases. WHO Technical Report Series* (2010).
18. May, M. *et al.* Prognosis of HIV-1 infected patients starting antiretroviral therapy in sub-Saharan Africa: a collaborative analysis of scaleup programmes. *Lancet* **376,** 449–457 (2011).

19. Collin, S. *et al.* Conflict and Kala-Azar: Determinants of Adverse Outcomes of Kala-Azar among Patients in Southern Sudan. *Clin. Infect. Dis.* **38,** 612–9 (2004).
20. Moons, K. G. M., Royston, P., Vergouwe, Y., Grobbee, D. E. & Altman, D. G. Prognosis and prognostic research: what, why, and how? *BMJ* **338,** 1317–1320 (2009).
21. Lyons, S., Veeken, H. & Long, J. Visceral leishmaniasis and HIV in Tigray, Ethiopia. *Trop. Med. Int. Health* **8,** 733–9 (2003).
22. Mueller, Y. *et al.* Risk factors for in-hospital mortality of visceral leishmaniasis patients in eastern Uganda. *Trop. Med. Int. Health* **14,** 910–7 (2009).
23. Seaman, J., Mercer, a J. & Sondorp, E. The epidemic of visceral leishmaniasis in western Upper Nile, southern Sudan: course and impact from 1984 to 1994. *Int. J. Epidemiol.* **25,** 862–71 (1996).
24. MSF. *Msf kala azar manual.* (2014).
25. Spiegelhalter DJ, Knill-Jones, R. Statistical and knowledge-based approaches to clinical decision-support systems, with an application in gastroenterology. *J. R. Stat. Soc.* **147,** 35–77 (1984).
26. Steyerberg, E. W. *Clinical Prediction Models: A Practical Approach to Development, Validation, and Updating. New York: Springer* (2009). doi:10.1017/CBO9781107415324.004

## 11. Annexes

### Annex 1: kala azar treatment card

Admission data entered ○
Exit data entered ○

**Kala Azar Treatment Card**    ***Treatment site: Abdurafi***

Name: ______________________________ Patient No.: __________ Lab No.: ________

Admission date: ________________ Sex: ______ (M/F) Age: ________ months/years

Current Address: Region: __________ Woreda: __________ Kebele: ____________
Migrant Worker / Resident / Settler / Other: ____________________
How long at current address: _____ years, _____ months
Origin (*if not resident*): Region: __________ Woreda: __________ Kebele: ____________

*Previous Kala Azar treatment:* ___ *(Y/N)* *Regime:* ______________________________

*Exit date:* ________________ *Location:* ______________________________

Sick for how long: _________ weeks/months    Weakness: ○ Collapse / ○ Severely weak / ○ Little or no

Weight: ________ kg Height: ________ cm Z-score (<19 yrs): _______ / BMI (≥19 yrs): _________

Spleen size: ___ cm Liver size: ___ cm Lymphadenopathy: __ (Y/N)

Hb: _______ g/dl / Clinical anaemia: ___ (Y/N)

Pregnancy: ___ (Y/N) Jaundice: ___ (Y/N) Oedema: ___ (Y/N)

| **Severity score** | |
|---|---|
| Age | |
| Weakness | |
| Nutritional status | |
| Hb | |
| **TOTAL** | |

Under TB Tx: __ (Y/N) VCT code: __ ART: __ (Y/N) FUCHIA#: _______ CD4 count: ________

Malaria RDT - Date: _______ Result: _____ (neg/pos)

IT Leish *(rK39)* - Date: ________ Result: _____ (neg/pos)

DAT - Date: ________ Result: _____ (neg/bl/pos) Titre (well): ______ (0-11)

Aspirate - Date: ________ Site: ○ Spleen / ○ LN / ○ BM Result: _____ (0-6)

Diagnosis: ○ PKA / ○ Relapse / ○ PKDL/PKMDL Treatment regimen: ____________________

Initial doses - SSG: ______ PM: ______ AmBisome (course): ________________ Miltefosine: ______

Ferrous fumarate/Folic acid: _____ tablets Folic acid: __ tablets PlumpyNut: _____ sachets

Vit A – Date: ____________ Dose: ________ IU Measles vaccination (<16 yrs) – Date: ____________

History:

______________________________________________________________
______________________________________________________________
______________________________________________________________
______________________________________________________________
______________________________________________________________

Temp: ________ °C RR: ________ /min PR: ________ /min BP: ______________ mmHg

| Date | Day of Tx | SSG dose | PM dose | AmB dose | Milt dose | FeFol | Folic acid | Complaints/complications<br>Other treatments | Temp | Weight (1x/week) | Bleeding? | Vomiting? | Diarrhoea? |
|---|---|---|---|---|---|---|---|---|---|---|---|---|---|
| | | | | | | | | | | | | | |
| | | | | | | | | | | | | | |
| | | | | | | | | | | | | | |
| | | | | | | | | | | | | | |
| | | | | | | | | | | | | | |
| | | | | | | | | | | | | | |
| | | | | | | | | | | | | | |
| | | | | | | | | | | | | | |
| | | | | | | | | | | | | | |
| | | | | | | | | | | | | | |
| | | | | | | | | | | | | | |
| | | | | | | | | | | | | | |
| | | | | | | | | | | | | | |
| | | | | | | | | | | | | | |
| | | | | | | | | | | | | | |
| | | | | | | | | | | | | | |
| | | | | | | | | | | | | | |
| | | | | | | | | | | | | | |
| | | | | | | | | | | | | | |
| | | | | | | | | | | | | | |
| | | | | | | | | | | | | | |
| | | | | | | | | | | | | | |
| | | | | | | | | | | | | | |
| | | | | | | | | | | | | | |

***Discharge summary***

Total amount of doses given – SSG: ________ PM: ________ AmBisome: ________ Miltefosine: ________

Complications during treatment: Occurrence of bleeding _____ (Y/N) Vomiting for more than 2 days in a row _____ (Y/N)

Any other complications or comments:

______________________________________________

______________________________________________

______________________________________________

TOC 1 - Date: _________ Site: ○ Spleen / ○ LN / ○ BM Result: ______ (0-6)

TOC 2 - Date: _________ Site: ○ Spleen / ○ LN / ○ BM Result: ______ (0-6)

TOC 3 - Date: _________ Site: ○ Spleen / ○ LN / ○ BM Result: ______ (0-6)

Exit – Date: ___________ Weight: _________ kg Spleen size: ______ cm Liver size: ______ cm

Lymphadenopathy: ___ (Y/N) Hb: ________ g/dl PK(M)DL lesions: ______________________________________

Final outcome: ○ Cured TOC neg / ○ Clinical cure / ○ Death / ○ Defaulter / ○ Transferred to _________________
